# Mechanism of Action of *Hedyotis diffusa* Extract in a Rat Model of Acute Lung Injury Based on Transcriptomic Analysis

**DOI:** 10.3390/biology15171549

**Published:** 2026-09-04

**Authors:** Chenyi Lu, Xinyi Yang, Xin Yang

**Affiliations:** School of Basic Medicine, Guizhou University of Traditional Chinese Medicine, Guiyang 550025, China; chen268695@163.com (C.L.); y2635248@163.com (X.Y.)

**Keywords:** *Hedyotis diffusa*, acute lung injury, inflammatory factors, transcriptomics, collaborative regulation

## Abstract

Objective: This study established a rat model of lipopolysaccharide (LPS)-induced acute lung injury (ALI) to evaluate pathological damage, collagen deposition, inflammatory cytokine levels, and key gene/protein expression following *Hedyotis diffusa* water extract (HDWE) intervention. Combined with ultra-high-performance liquid chromatography-quadrupole Orbitrap high-resolution mass spectrometry (UHPLC-Q-Orbitrap HRMS), transcriptomic analysis, and molecular simulation, this study identified the bioactive components of HDWE, evaluated their potential interactions with ALI-related targets, and explored the multi-omics-based protective mechanisms of HDWE. Methods: Thirty-six Sprague–Dawley (SD) rats were randomly divided into six groups: Control group, ALI group, DXMS group, HDWE-L group (100 mg/kg), HDWE-M group (200 mg/kg), and HDWE-H group (300 mg/kg). Hematoxylin and eosin (H&E) and Masson’s trichrome staining were used to evaluate lung pathological changes and collagen deposition. Enzyme-linked immunosorbent assay (ELISA) was used to measure serum tumor necrosis factor-α TNF−α interleukin-1β IL−1β, erleukin-6 (IL-6), and interleukin-10 (IL-10) levels. Transcriptomic analysis identified differentially expressed genes (DEGs), followed by Gene Ontology (GO), Kyoto Encyclopedia of Genes and Genomes (KEGG), receiver operating characteristic (ROC), and immune infiltration analyses. Quantitative real-time polymerase chain reaction (qRT-PCR) detected the mRNA expression levels of *SPHK1*, *RELA*, and *NFKBIA*. Immunohistochemistry evaluated the expression of eight hub targets, including endothelin-1 (EDN1), sphingosine kinase 1 (SPHK1), intercellular adhesion molecule 1 (ICAM1), interleukin-17 (IL-17), prostaglandin-endoperoxide synthase 2 (PTGS2/COX-2), NF-κB p65 (encoded by *RELA*), WT1-associated protein (WTAP), and myeloperoxidase (MPO). UHPLC-Q-Orbitrap HRMS characterized HDWE constituents. Molecular docking analysis was performed between 22 compounds and eight hub targets, followed by 100 ns molecular dynamics simulations and molecular mechanics-Poisson–Boltzmann surface area (MM/PBSA) binding free energy calculations for five core targets. Compared with the control group, the ALI group showed increased levels of TNF-α (86%), IL-1β (107%), and IL-6 (66%), accompanied by a 43% reduction in IL-10 and a 300% increase in lung collagen deposition. All HDWE doses alleviated inflammatory responses, with medium-dose HDWE showing the most pronounced effects. Specifically, medium-dose HDWE increased IL-10 levels by 52% and reduced IL-6, TNF-α, and IL-1β levels by 18%, 22%, and 11%, respectively. Transcriptomic analysis identified 2512 DEGs between the control group and ALI groups, 832 exclusive DEGs between the ALI group and HDWE-M groups, and 876 overlapping DEGs enriched in TNF, IL-17, and NF-κB signaling pathways. The eight-hub-gene diagnostic model achieved an area under the curve (AUC) of 0.969. *RELA*, *SPHK1*, and four other hub genes showed positive correlations with Th1, Th17, and neutrophil infiltration. In the ALI group, *SPHK1*, *RELA*, and *NFKBIA* mRNA expression levels were 1.30-, 0.96-, and 0.71-fold of those in the control group, respectively. Compared with the ALI group, high-dose HDWE treatment and low-dose HDWE treatment reduced *SPHK1* expression to 0.62- and 0.57-fold, respectively, and increased *NFKBIA* expression to 1.68- and 1.58-fold, respectively. High-dose HDWE treatment reduced *RELA* expression to 0.43-fold. The expression levels of inflammation-related proteins were increased in the ALI group and were reduced after HDWE treatment. Twenty-two HDWE components were identified, 16 of which met the docking criteria. Asperulosidic acid exhibited favorable predicted binding affinities with all eight targets, with calculated binding free energies of −14.74, −14.92, −17.58, −23.04, and −16.10 kcal/mol for MPO, IL-17, NF-κB p65, PTGS2/COX-2, and SPHK1, respectively. Conclusions: This study provides systematic in vivo pharmacodynamic and in silico component-target evidence regarding the protective effects of HDWE against LPS-induced ALI. HDWE treatment increased *NFKBIA* expression and reduced *SPHK1*, *RELA*, and multiple inflammatory protein levels, suggesting that HDWE may regulate the IL-17/NF-κB-associated inflammatory network, although direct causal relationships require further validation. Asperulosidic acid may represent a key bioactive component with broad target-binding potential. This study was limited by the use of an LPS-induced rat ALI model without gene knockout or target inhibitor validation; therefore, further functional experiments are required to confirm the proposed regulatory mechanisms.

## Simple Summary

Acute lung injury has a high mortality rate, and its pathogenesis involves excessive inflammation, oxidative stress imbalance, and alveolar barrier disruption. As a traditional Chinese medicine, *Hedyotis diffusa* shows potential protective effects against acute lung injury; however, its specific molecular mechanisms remain unclear. In this study, we established a lipopolysaccharide (LPS)-induced rat model of acute lung injury and, using transcriptomic analysis, identified differentially expressed genes and key signaling pathways following drug intervention. The binding affinities between the active compounds and the core targets were further evaluated using molecular docking. We explored the potential molecular mechanisms by which *Hedyotis diffusa* alleviates lung injury from multiple perspectives, providing experimental evidence and theoretical insights for further mechanistic studies and clinical drug development.

## 1. Introduction

Acute lung injury (ALI) is characterized by disruption of the alveolar epithelial and pulmonary microvascular endothelial barriers. Increased vascular permeability promotes protein leakage and inflammatory cell infiltration, leading to diffuse pulmonary interstitial and alveolar edema—an important early event in the development of acute respiratory distress syndrome (ARDS) [1,2,3]. Following barrier disruption, endothelial cells release abundant pro-inflammatory mediators, forming a complex cytokine network. Circulating neutrophils undergo chemotaxis, adhere to the endothelium, and migrate into the lungs, where they release proteases and reactive oxygen species that amplify local inflammation, further damaging alveolar structures and promoting ALI progression [4]. Clinically, ALI patients present with progressive hypoxemia and dyspnea; persistent deterioration often leads to severe ARDS with persistently high mortality [5]. Current management remains largely supportive, relying on broad-spectrum anti-inflammatory strategies, as specific agents that effectively target inflammatory cascades and preserve endothelial barrier function are lacking [6]. Uncontrolled inflammatory activation is widely recognized as a central driver of ALI pathogenesis, involving excessive inflammatory signaling, apoptosis, oxidative stress, and coagulation dysfunction [7,8,9]. Compared with synthetic drugs, natural plant extracts offer multi-target regulatory effects and favorable safety profiles, making them attractive candidates for ALI prevention and treatment [10]. Previous studies have shown that several natural compounds, including *Panax notoginseng* saponins, *Perilla frutescens* extracts, and atractylodin, alleviate LPS-induced lung injury by modulating NF-κB and NLRP3 inflammatory pathways [11,12,13]. Consequently, identifying bioactive components from traditional Chinese medicine and elucidating their mechanisms of action represent important directions in ALI research.

*Hedyotis diffusa* Willd. (HDW), a traditional Chinese medicinal herb of the Rubiaceae family, has long been used for pulmonary inflammatory disorders [14]. Phytochemical studies have shown that HDW is rich in iridoid glycosides and flavonoids, including asperulosidic acid, asperuloside, luteolin, and quercetin [14]. HDW exhibits anti-inflammatory, antioxidant, and immunomodulatory activities, suggesting potential protective effects against pulmonary inflammation [15,16]. Preclinical studies have confirmed that HDW-derived preparations alleviate LPS-induced systemic and pulmonary inflammatory injury [17,18,19].

However, existing reports are largely phenotypic, with limited transcriptomic profiling and molecular target validation. To address this gap, we established an LPS-induced rat ALI model and performed transcriptomic analysis to identify differentially expressed genes and key pathways affected by HDWE intervention. We also combined molecular docking to evaluate potential interactions between HDWE-derived compounds and inflammation-related molecular targets. This study aimed to elucidate the molecular mechanisms underlying the protective effects of HDWE against ALI, providing experimental evidence for further mechanistic and therapeutic studies.

## 2. Materials and Methods

### 2.1. Experimental Medicinal Materials

*Hedyotis diffusa* was purchased from Sichuan Qianfang Chinese Medicine Co., Ltd, Chengdu, China, and authenticated by Professor Yang Xin from Guizhou University of Traditional Chinese Medicine. For decoction preparation, 300 g of *Hedyotis diffusa* was rinsed with tap water and soaked in 2000 mL of distilled water for 30 min. The mixture was then heated to boiling in a decoction device and maintained at a gentle boil for 30 min. The decoction was filtered and transferred into a clean container. An additional 1000 mL of distilled water was added to the herb residue, and the extraction procedure was repeated. The two filtrates were combined and concentrated to a final concentration equivalent to 1 g of crude drug per mL [20]. The prepared HDWE was stored at 4 °C until further use for in vivo intervention experiments in rats. In addition, an aliquot of the same batch of HDWE was concentrated using a rotary evaporator and subsequently lyophilized to obtain HDWE powder. The lyophilized powder was stored at −80 °C for subsequent chemical composition analysis using UHPLC-Q-Orbitrap HRMS (Thermo Fisher Scientific, Bremen, Germany). Lipopolysaccharide (LPS) was obtained from Sigma-Aldrich (St. Louis, MO, USA), and dexamethasone sodium phosphate injection was purchased from Tianjin Pharmaceutical Group Jiaozuo Co., Ltd., Jiaozuo, China.

### 2.2. ALI Model Establishment and Drug Intervention

Thirty-six SPF-grade Sprague–Dawley (SD) rats (18 males and 18 females), weighing 200 ± 20 g, were obtained from the Laboratory Animal Institute of the university (license No. SYXK (Qian) 2021-0003). The rats were housed in an SPF-grade animal facility under controlled conditions (temperature: 22–25 °C; humidity: 50–60%; 12 h light/dark cycle) and allowed to acclimatize for 1 week before experimentation. The rats were randomly divided into six groups stratified by sex (*n* = 6 per group): Control group, ALI group, DXMS group, HDWE-L group, HDWE-M group, and HDWE-H group. Except for the control group, which received an equal volume of normal saline by intraperitoneal injection, all other groups were intraperitoneally injected with LPS solution (5 mg/kg) to establish the ALI model. After 24 h, rats in the Control group and ALI group received normal saline by gavage. Rats in the DXMS group received dexamethasone (2.5 mg/kg) by gavage, whereas rats in the HDWE-L, HDWE-M, and HDWE-H groups received HDWE at doses of 100, 200, and 300 mg/kg, respectively. All treatments were administered once daily for 7 consecutive days. All HDWE doses were expressed as crude herbal material equivalents.

Before sample collection, all rats were subjected to terminal anesthesia via intraperitoneal injection of urethane (1.6 g/kg). No postoperative survival procedures were performed in this study. A standardized animal welfare assessment protocol was implemented throughout the experiment. The activity status, food intake, and behavioral changes in the rats were monitored daily, and pain and stress levels were evaluated. When animals reached humane endpoint criteria [21], euthanasia was performed by overdose anesthesia. After 24 h of fasting following the final administration, rats were anesthetized with urethane. Blood samples were collected from the abdominal aorta for serum separation, and lung tissues were harvested for subsequent analyses.

### 2.3. Pulmonary Histopathological Analysis

After anesthesia, the lower lobe of the right lung was dissected and fixed in 4% paraformaldehyde. After 48 h of fixation, tissues underwent graded ethanol dehydration, xylene clearing, and overnight paraffin embedding. Paraffin blocks were serially sectioned into 4 μm-thick sections and subjected to routine hematoxylin and eosin (H&E) staining using an H&E staining kit (Cat. No. G1120-100, Solarbio Science & Technology Co., Ltd., Beijing, China). Histopathological changes in lung tissues were observed under an optical microscope. A blinded semi-quantitative five-point scoring system was applied to evaluate lung injury based on four indicators: inflammatory cell aggregation in alveolar and capillary walls, alveolar septal thickening and hyaline membrane formation, alveolar congestion, and pulmonary hemorrhage. Each indicator was independently scored from 0 to 4, representing pathological changes ranging from normal tissue to severe injury. The total pathological score was calculated by summing individual scores (range: 0–16), with higher scores indicating more severe inflammatory infiltration, congestion, hemorrhage, and structural damage in lung tissues.

### 2.4. Masson’s Trichrome Staining of Rat Lung Tissue

Paraffin-embedded lung tissues were sectioned into 4 μm-thick sections, deparaffinized, and rehydrated. The sections were incubated with Bouin’s solution overnight at room temperature. Thorough rinsing was performed under running water until the yellow coloration was completely removed. Nuclear staining, muscle fiber staining, and collagen fiber staining were sequentially performed using a Masson’s trichrome staining kit (Cat. No. G1340, Solarbio Science & Technology Co., Ltd., Beijing, China) according to the manufacturer’s instructions. The sections were dehydrated and mounted with neutral balsam. Five non-overlapping high-power fields (×200) were randomly selected for imaging. Images were analyzed using Aipathwell^®^ software v2 (Servicebio, Wuhan, China). The collagen-positive area ratio was calculated using the following formula: Collagen-positive area ratio (%) = (collagen-positive staining area/total lung tissue area in the analyzed field) × 100%. A higher percentage indicated greater collagen deposition and pulmonary fibrosis.

### 2.5. Detection of Serum Inflammatory Cytokines

Serum levels of IL-6 (Cat. No. ZK-R3141, Shenzhen Zike Biotechnology Co., Ltd.; Shenzhen, China), IL-10 (Cat. No. ZK-R3145, Shenzhen Zike Biotechnology Co., Ltd.; Shenzhen, China), IL-1β (Cat. No. ZK-R3370, Shenzhen Zike Biotechnology Co., Ltd.; Shenzhen, China) and TNF-α (Cat. No. ZK-R3528, Shenzhen Zike Biotechnology Co., Ltd.; Shenzhen, China) were measured by ELISA assays following the manufacturer’s instructions.

### 2.6. Transcriptomic Analysis

#### 2.6.1. Sample Preparation and RNA Sequencing

Four rats were randomly selected from the control group, ALI group, and HDWE-M group. Lung tissues were collected into cryopreservation tubes and stored at −80 °C until further analysis. Total RNA was extracted from lung tissues using the TRIzol method. cDNA libraries were constructed and purified according to previously described protocols [22], including mRNA enrichment with Oligo(dT), mRNA fragmentation, reverse transcription into cDNA, adapter ligation, fragment size selection, and PCR amplification of size-selected products. Sequencing was performed using the NovaSeq X Plus platform (Illumina NovaSeq 6000 sequencing platform) (Illumina, San Diego, CA, USA).

#### 2.6.2. Data Quality Control and Screening of Differentially Expressed Genes

Raw sequencing reads containing a high proportion of unknown bases (N), excessively short sequences, low-quality reads, or adapter contamination were filtered using fastp software (v0.23.2, https://packages.guix.gnu.org/packages/fastp/0.23.2/ (accessed on 10 June 2025)) to obtain high-quality clean reads. The clean reads were aligned to the reference genome using HISAT2 software v2.2.1 (https://daehwankimlab.github.io/hisat2/download/ (accessed on 10 June 2025)). Gene expression levels were quantified using RSEM software v1.3.3 (http://deweylab.github.io/RSEM/ (accessed on 10 June 2025)). Differentially expressed genes (DEGs) were identified using DESeq2 software v1.52.0 (https://github.com/thelovelab/DESeq2 (accessed on 10 June 2025)) with the screening criteria of false discovery rate (FDR) < 0.05 and |log2FC| ≥ 1.

#### 2.6.3. Data Analysis

Clustering analysis was performed based on the identified DEGs, and the results were visualized using volcano plots to display the distribution of DEGs and expression differences among groups. Shared DEGs among different comparisons were analyzed using Venn diagrams. Kyoto Encyclopedia of Genes and Genomes (KEGG) pathway enrichment analysis was performed using Fisher’s exact test, with *p* < 0.05 considered statistically significant [23,24].

### 2.7. ROC Curve Analysis of Hub Genes

Receiver operating characteristic (ROC) curves for hub genes were generated using R software (v4.3.0) based on the GSE3077, GSE40885, and GSE5272 datasets. The area under the ROC curve (AUC) for individual hub genes was calculated to evaluate their potential diagnostic performance for ALI [25].

### 2.8. Immune Cell Infiltration Analysis of Hub Genes

Immune cell infiltration datasets, including 28 immune cell types classified into adaptive and innate immune categories, were obtained from TISIDB (https://cis.hku.hk/TISIDB/download.php (accessed on 10 June 2025)). Visualization of correlations between hub gene expression and immune cell infiltration patterns was performed using relevant R software packages based on immune infiltration data and merged GEO datasets [26].

### 2.9. Immunohistochemical Staining of MPO

Fixed lung tissues were sectioned into 4 μm-thick slices, followed by deparaffinization and rehydration. Antigen retrieval was performed using 20× Tris-EDTA antigen retrieval buffer (pH 8.0) in an antigen retrieval instrument (ARI-4, Servicebio, Wuhan, China) with heat treatment for 15 min. After cooling to room temperature, sections were washed three times in PBS on a shaker for 5 min each. Endogenous peroxidase activity was blocked by incubation with 3% hydrogen peroxide solution at room temperature in the dark for 25 min, followed by PBS washing. Sections were blocked at room temperature for 30 min. After removal of the blocking solution, MPO primary antibody (GB11224, Solarbio Science & Technology Co., Ltd., Beijing, China) diluted at 1:2000 was added and incubated overnight at 4 °C. Sections were subsequently incubated with HRP-conjugated goat anti-rabbit secondary antibody (G1302, Servicebio) at room temperature for 50 min. After washing, freshly prepared DAB chromogenic solution was applied, and color development was monitored under a microscope. The reaction was terminated by rinsing sections with tap water. Subsequent procedures included nuclear counterstaining, dehydration, clearing, and mounting with neutral balsam. The immunohistochemical images were imported into Aipathwell^®^ digital pathology analysis software (Servicebio, Wuhan, China) for quantitative analysis [27]. Since MPO is a specific marker of neutrophils, MPO-positive cells were used to evaluate neutrophil infiltration in lung tissues. The software automatically identified MPO-positive cells and quantified the numbers of MPO-positive cells and total cells. The MPO-positive cell ratio was calculated using the following formula: MPO-positive cell ratio (%) = (number of MPO-positive cells/total number of cells) × 100%. Neutrophil infiltration was evaluated based on the proportion of MPO-positive cells.

### 2.10. Immunohistochemical Detection of Core Targets in Lung Tissue

Lung tissue samples were fixed with 4% paraformaldehyde solution prepared in 0.1 mol/L PBS containing 0.1% DEPC (pH 7.0–7.6). After dehydration, embedding, sectioning, heat-induced antigen retrieval, and blocking, primary antibodies against EDN1 (Cat. No. BS-0954R, Bioss, Beijing, China), WTAP (Cat. No. DF3282, Proteintech, Wuhan, China), IL-17 (Cat. No. AB214588, Abcam, Cambridge, UK), PTGS2/COX-2 (Cat. No. BS-0732R, Bioss, Beijing, China), ICAM1 (Cat. No. BS-4617R, Bioss, Beijing, China), and NF-κB p65 (Cat. No. BS-0456R, Bioss, Beijing, China) were applied at 1:200 and incubated overnight at 4 °C. After washing, corresponding secondary antibodies were added and incubated for 50 min at room temperature. DAB chromogenic development, hematoxylin counterstaining, dehydration, clearing, and mounting were subsequently performed. Images were captured using a light microscope (LSM 880, Zeiss, Oberkochen, Germany). ImageJ software (v1.54f) was used to quantify the integrated optical density (IOD) of positive staining, reflecting the relative expression levels of EDN1, WTAP, IL-17, PTGS2/COX-2, ICAM1, and NF-κB p65 proteins among different groups.

### 2.11. UPLC-Q-Orbitrap HRMS Identification of HDWE Components

The chemical constituents of HDWE were characterized according to a previously established and published UHPLC-Q-Orbitrap HRMS method developed by our research team [28]. Briefly, 100 mg of lyophilized HDWE powder was accurately weighed and extracted with 500 μL of methanol–water solvent (4:1, *v*/*v*). The samples were centrifuged at 12,000 rpm for 15 min at 4 °C, and the supernatants were collected and filtered through a 0.22 μm microporous membrane to obtain the sample solutions for UHPLC-Q-Orbitrap HRMS analysis. Chromatographic separation was performed using a Vanquish ultra-high-performance liquid chromatography system equipped with an Acquity UPLC BEH C18 column (100 mm × 2.1 mm, 1.7 μm). The injection volume was set at 5 μL, and the flow rate was maintained at 0.5 mL/min. The mobile phases consisted of ultrapure water containing 0.1% formic acid (phase A) and acetonitrile containing 0.1% formic acid (phase B). The gradient elution program was performed according to the procedure described in Supplementary Table S1. Mass spectrometric data were acquired using a Q Exactive Focus (Thermo Fisher Scientific, Waltham, MA, USA) high-resolution mass spectrometer equipped with a FullScan ddMS2 acquisition mode. The chemical constituents of HDWE were simultaneously detected in both positive and negative ionization modes. The major MS parameters were set as follows: sheath gas flow rate, 45 Arb; auxiliary gas flow rate, 15 Arb; capillary temperature, 400 °C; full MS resolution, 70,000; MS/MS resolution, 17,500; stepped normalized collision energies (NCE), 15, 30, and 45. The spray voltage was set at 4.0 kV in positive ion mode and −3.6 kV in negative ion mode.

### 2.12. Molecular Docking Analysis

The interactions between HDWE compounds and hub targets were analyzed using the molecular docking module of SYBYL 2.1.1. The docking results were evaluated using the total score (T_Score) as the screening criterion [29]. Crystal structures of EDN1 (PDB ID: 8HCQ), SPHK1 (PDB ID: 3VZD), ICAM1 (PDB ID: 7BG7), IL-17 (PDB ID: 9SQX), PTGS2/COX-2 (PDB ID: 5F19), RELA (PDB ID: 7LF4), WTAP (PDB ID: 7VF2), and MPO (PDB ID: 5FIW) were obtained from the RCSB Protein Data Bank (https://www.rcsb.org/ (accessed on 10 June 2025)).

### 2.13. Molecular Dynamics Simulation and MM-PBSA Binding Free Energy Calculation

Molecular dynamics simulations of protein–ligand complexes were performed using GROMACS 2022.2 software. The Amber99SB-ILDN force field was applied to proteins, and ligand topology files were generated using GAFF2 through Sobtop (v1.0 (dev3.1)). Partial charges were assigned using the RESP method. The systems were solvated using the TIP3P water model, and 0.15 mol/L NaCl was added to maintain physiological ionic strength and electroneutrality. Long-range electrostatic interactions were calculated using the particle mesh Ewald (PME) method, with a non-bonded interaction cutoff of 1.2 nm and an integration timestep of 2 fs. Following two-step energy minimization, 1 ns NVT (300 K) and 1 ns NPT (1 bar) restrained equilibration simulations were sequentially performed, followed by unrestrained production simulations. System stability was evaluated using root-mean-square deviation (RMSD), root-mean-square fluctuation (RMSF), radius of gyration (Rg), solvent-accessible surface area (SASA), hydrogen bond analysis, and free energy landscape analysis. Finally, MM/PBSA binding free energies were calculated using gmx_MMPBSA (v1.6.4, MMPBSA.py v.16.0) to evaluate the predicted binding affinities between asperulosidic acid and the target proteins SPHK1, IL-17, PTGS2/COX-2, RELA, and MPO.

### 2.14. RT-qPCR Detection of SPHK1 and NFKBIA mRNA Expression

Quantitative real-time PCR (RT-qPCR) was performed to detect the relative mRNA expression levels of the pathway-related genes *SPHK1*, *RELA*, and *NFKBIA* in lung tissues. Total RNA was extracted from lung tissues of each group and reverse-transcribed into cDNA. *GAPDH* was used as the internal reference gene during amplification. Relative mRNA expression levels were calculated using the 2^−ΔΔCt^ method. Three technical replicates were performed for each group to reduce experimental variation. Primer sequences, amplified fragment lengths, and annealing temperatures of target genes and the reference gene are listed in Table S2.

### 2.15. Statistical Analysis

Measurement data conforming to a normal distribution were expressed as “mean ± standard deviation”. Normally distributed data were analyzed via one-way ANOVA followed by LSD-t post hoc test for pairwise comparisons; non-normal pathological score data were analyzed using the Kruskal–Wallis test. *p* < 0.05 was defined as statistically significant.

## 3. Results

### 3.1. Gross Observation, Histopathological and Fibrosis Staining Evaluation of Rat Lung Tissues

**Figure 1:**
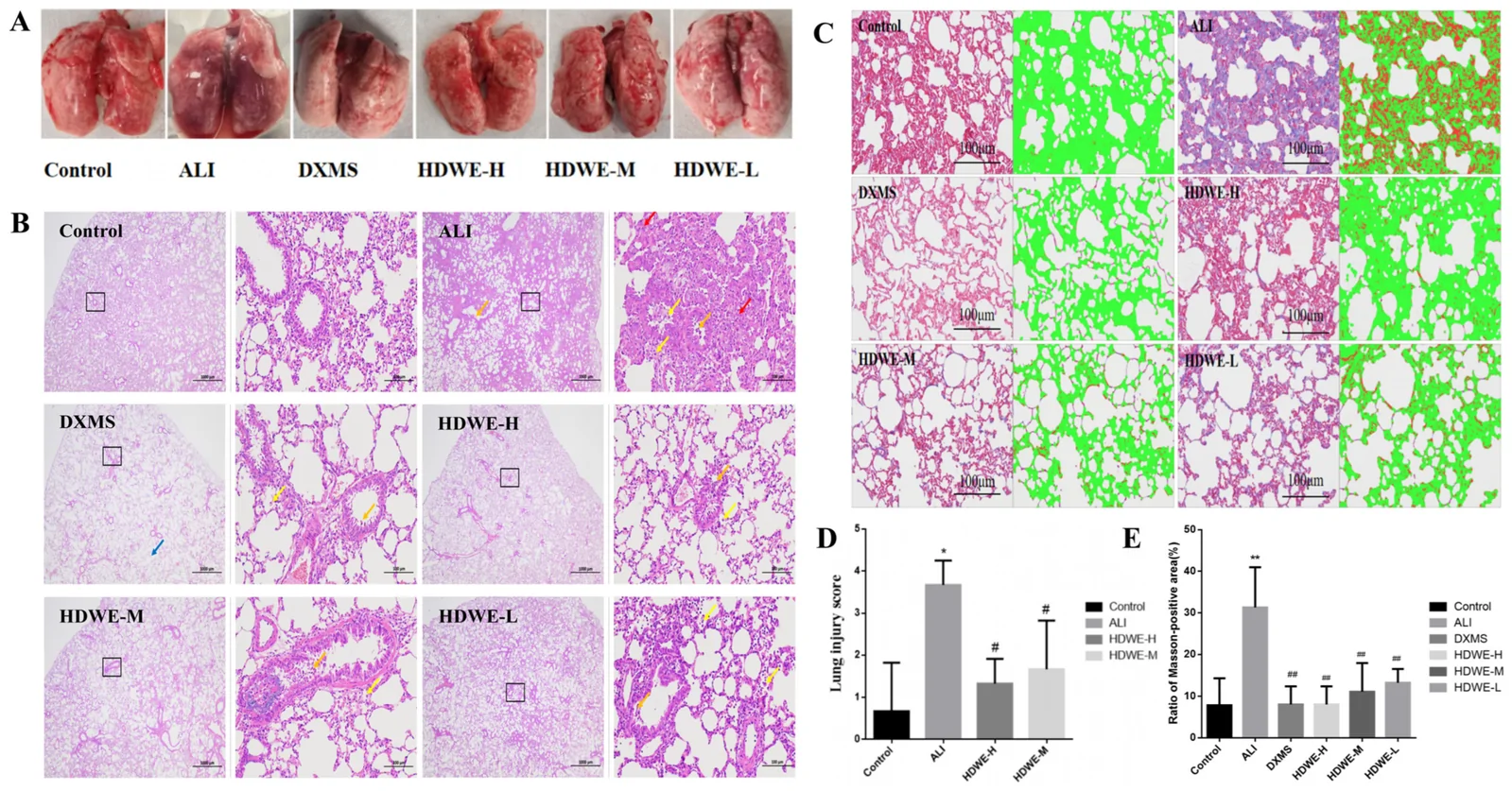
Gross appearance, histopathological lesions, and collagen deposition in lung tissues of each rat group. (**A**) Gross macroscopic observation of intact lung tissues from the Control, ALI, DXMS, HDWE-H, HDWE-M, and HDWE-L groups. (**B**) Hematoxylin and eosin (H&E) staining for histopathological assessment of lung injury. Images were captured under 20× and 200× magnification. Black squares denote the localized fields for high-power magnification. Arrows indicate typical pathological lesions: red arrows indicate granulocyte infiltration in alveolar walls, focal thickening of alveolar walls, and widened alveolar septa; blue arrows represent alveolar ectasia, uneven alveolar size, and stenosis of surrounding alveoli; orange arrows indicate exfoliated cells inside the bronchioles. (**C**) Representative Masson-stained sections and AI segmentation pseudo-color images processed using Aipathwell^®^ software. The left panels show original Masson-stained sections, and the right panels show AI-segmented images. In pseudo-color images, red indicates collagen-positive areas, green represents lung tissue background, and white denotes alveolar cavities. In original stained sections, collagen fibers are stained blue, while muscle fibers and cytoplasm appear red. Scale bar = 100 μm. (**D**) Pathological scores of lung tissues. * *p* < 0.05 versus Control group; ^#^ *p* < 0.05 versus ALI group. (**E**) Quantitative analysis of the percentage of collagen-positive ratio. ** *p* < 0.01 versus Control group; ^##^ *p* < 0.01 versus ALI group.

Macroscopic observation of lung tissues revealed reddish and homogeneous lungs in the Control group without congestion, edema, or petechiae. In contrast, marked pulmonary congestion and swelling, accompanied by extensive punctate hemorrhage and consolidation lesions, were observed in the ALI group. Treatment with DXMS and serially graded doses of HDWE alleviated pulmonary congestion, edema, and pathological injury to varying degrees (Figure 1A). As illustrated in Figure 1B, the lung surface in the Control group was covered with intact and smooth serosa without obvious abnormalities. The lung parenchyma contained hierarchically branched intrapulmonary bronchi and abundant terminal alveoli. Bronchial architecture remained intact, and alveolar walls consisted of clear monolayer epithelium with negligible inflammatory infiltration. Compared with the Control group, the normal structures of bronchi and alveoli were disrupted in the ALI group. Slightly shed cells were observed within bronchioles (orange arrows), while granulocyte infiltration and moderate thickening of alveolar septa emerged in alveolar walls. Sporadic foamy cells accumulated in bronchioles and alveoli (yellow arrows). The severity of pulmonary pathological damage was reduced to varying degrees in the DXMS, HDWE-H, HDWE-M, and HDWE-L groups compared with the ALI group. Although DXMS and HDWE-L showed partial pathological improvement, obvious individual variation and large standard deviations were observed in these two groups. Therefore, statistical analysis of pathological scores shown in Figure 1E was only performed among the Control, ALI, HDWE-H, and HDWE-M groups. The Kruskal–Wallis test indicated significant overall differences among these four groups.

Masson’s trichrome staining was performed to evaluate interstitial collagen deposition in rat lung tissues (Figure 1C). Intact alveolar architecture and thin alveolar walls with scattered purple-blue collagen fibers in the interstitium were observed in the Control group. Diffuse thickening of alveolar walls and massive abnormal accumulation of interstitial collagen fibers occurred in the ALI group. Fibrotic lesions were alleviated to different extents after intervention with DXMS and high-, medium-, and low-dose HDWE. Figure 1E presents quantitative data for the collagen-positive area ratio. The collagen-positive area ratio in the ALI group was approximately four-fold higher than that in the Control group. Compared with the ALI group, DXMS, HDWE-H, HDWE-M, and HDWE-L reduced the collagen-positive area ratios by 74.38%, 73.39%, 64.59%, and 57.66%, respectively, with statistically significant differences (*p* < 0.01).

### 3.2. Changes in Serum Inflammatory Cytokines

**Figure 2:**
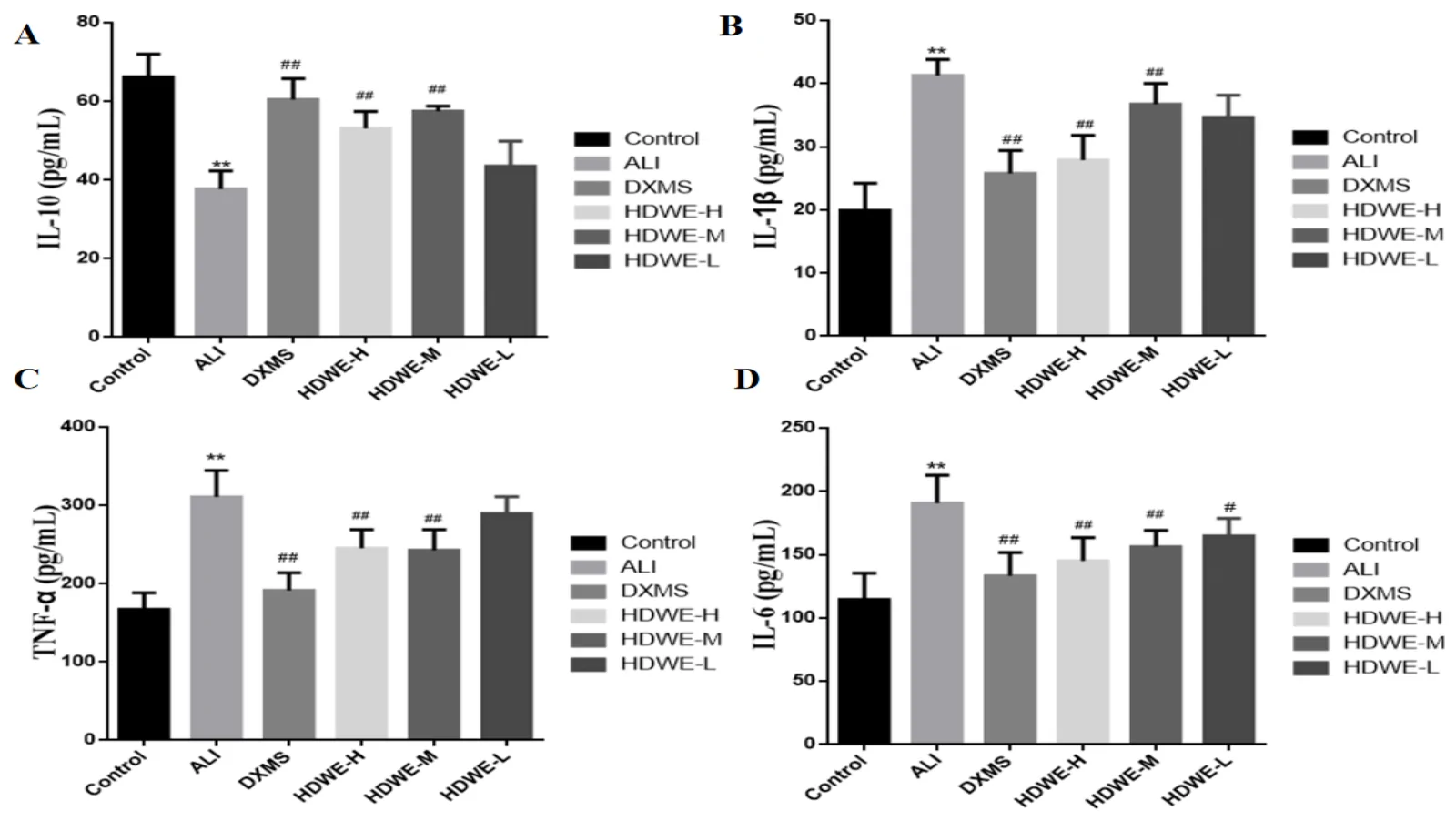
Detection of serum inflammatory cytokine levels in each group. (**A**) IL-10. (**B**) IL-1β. (**C**) TNF-α. (**D**) IL-6. Data are presented as mean ± SD. ** *p* < 0.01 versus the Control group. ^#^ *p* < 0.05 ^##^ *p* < 0.01 versus ALI group.

As shown in Figure 2, compared with the Control group, the serum levels of pro-inflammatory cytokines IL-6, TNF-α, and IL-1β in the ALI group were increased by approximately 66%, 86%, and 107%, respectively, whereas the level of the anti-inflammatory cytokine IL-10 was decreased by 43%. All differences were statistically significant (*p* < 0.01). DXMS and graded doses of HDWE (high-, medium-, and low-dose HDWE) alleviated the inflammatory imbalance. Specifically, medium-dose HDWE exhibited the optimal efficacy, increasing IL-10 levels by 54% and reducing IL-6, TNF-α, and IL-1β levels by 18%, 22%, and 11%, respectively, compared with the ALI group. Significant improvements were also observed in the high-dose and low-dose HDWE groups to different extents. (*p* < 0.05, *p* < 0.01).

### 3.3. Transcriptomic Profiling

**Figure 3:**
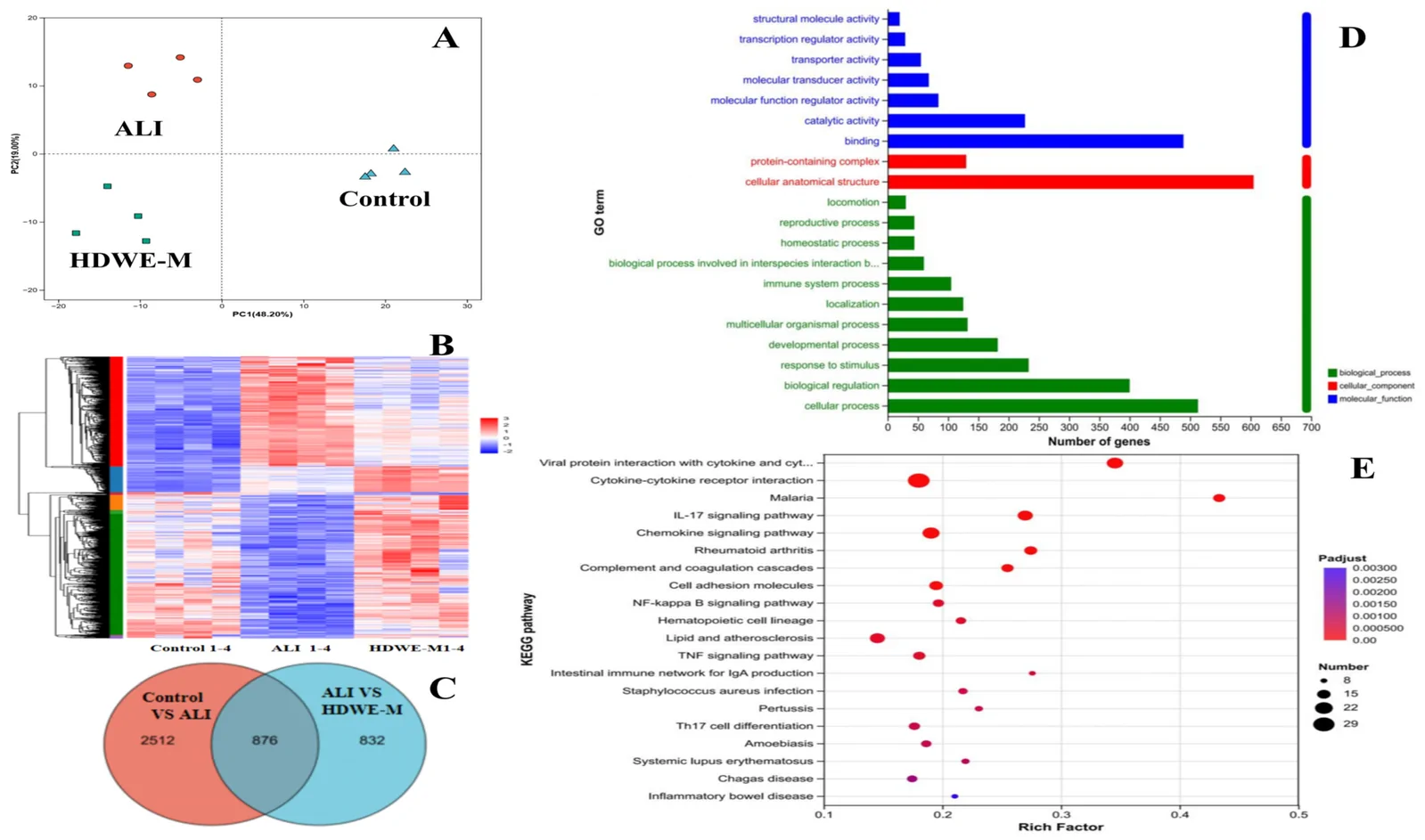
Bioinformatics analysis of differentially expressed genes. (**A**) Principal component analysis (PCA) of samples showing good intragroup consistency and intergroup separation. (**B**) Heatmap illustrating the expression patterns of DEGs across all samples. (**C**) Venn diagram showing the overlap of DEGs among different comparison groups. (**D**) GO functional enrichment bubble plot presenting the significantly enriched functional terms of DEGs. (**E**) KEGG pathway enrichment analysis displaying the key signaling pathways enriched by DEGs.

Transcriptomic analysis was performed on lung tissues from rats in the Control, ALI, and HDWE-M groups. PCA results (Figure 3A) showed clear clustering of samples within each group, distinct separation among groups, and good sample repeatability, indicating successful model establishment and drug intervention. The gene expression clustering heatmap (Figure 3B) directly reflected differences in gene expression profiles among groups. Statistical analysis identified 2512 unique differentially expressed genes (DEGs) between the Control and ALI groups, 832 unique DEGs between the ALI and HDWE-M groups, and 876 intersecting DEGs between the two comparisons (Figure 3C). The pathway analysis of DEGs is presented in Tables S3 and S4. GO functional enrichment analysis (Figure 3D) revealed that DEGs were mainly enriched in inflammation- and injury-related terms, including cellular processes, response to stimuli, binding functions, and cellular components. KEGG pathway enrichment analysis (Figure 3E) identified core inflammatory pathways, including cytokine–cytokine receptor interaction, TNF signaling pathway, IL-17 signaling pathway, and NF-κB signaling pathway. Key genes involved in these pathways included *RELA*, *SPHK1*, *IL17A*, *ICAM1*, *EDN1*, and *PTGS2* (COX-2). Pathways related to acute lung injury are presented in Table S5.

### 3.4. Differential Gene ROC and Immune Infiltration Analysis

**Figure 4:**
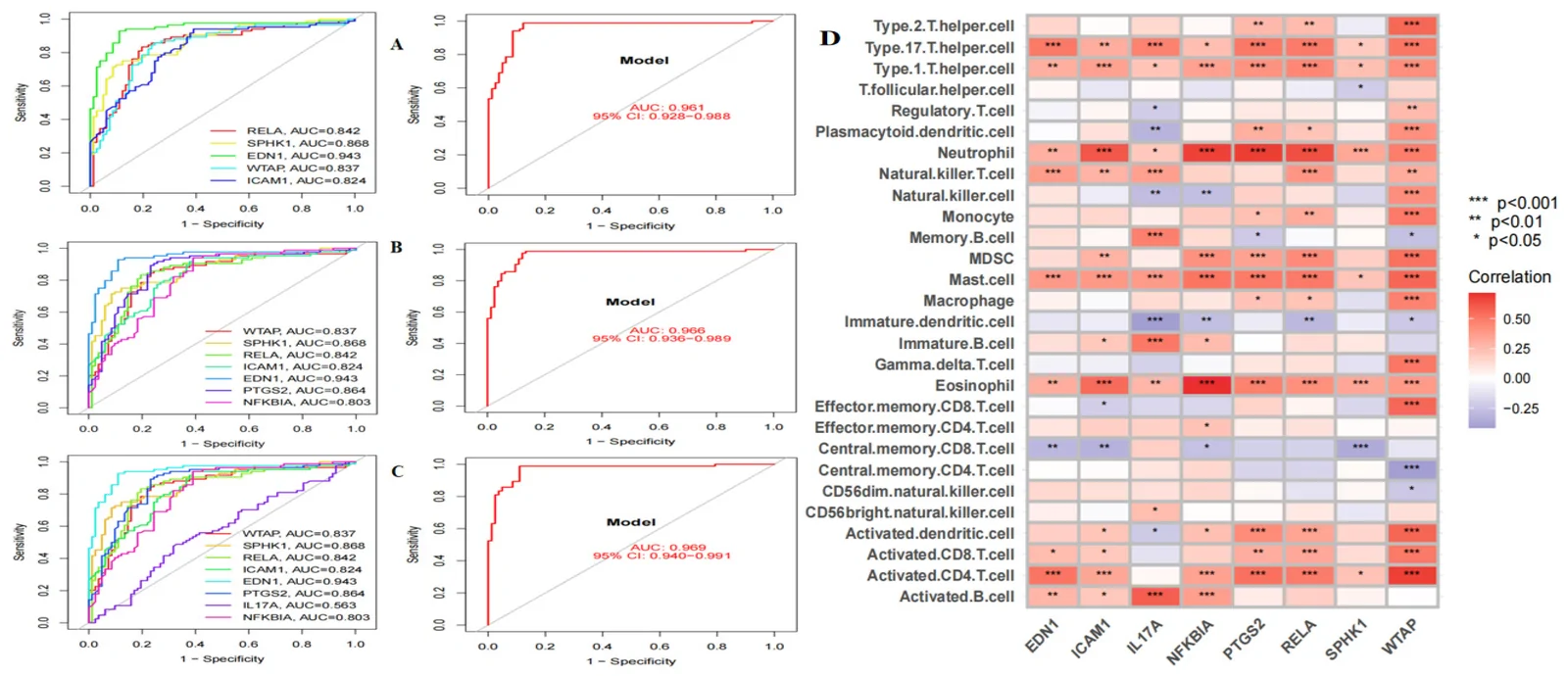
ROC curve analysis and immune cell infiltration correlation of ALI-related hub genes. (**A**) ROC curves of the five hub genes (*RELA*, *SPHK1*, *EDN1*, *WTAP*, and *ICAM1*) and their combined model, AUC = 0.961 (95% CI: 0.928–0.988). (**B**) ROC curves of seven hub genes (*WTAP*, *SPHK1*, *RELA*, *ICAM1*, *EDN1*, *PTGS2*, and *NFKBIA*) and their combined model, AUC = 0.966 (95% CI: 0.936–0.989). (**C**) ROC curves of all eight screened hub genes and the final combined model, AUC = 0.969 (95% CI: 0.940–0.991). The grey diagonal line indicates the reference line for random classification (AUC = 0.5). AUC, area under the ROC curve; CI, confidence interval. (**D**) The horizontal axis represents screened hub genes, and the vertical axis denotes 28 types of immune infiltrating cells. The color gradient indicates the Pearson correlation coefficient. Asterisks represent statistical significance: *** *p* < 0.001, ** *p* < 0.01, * *p* < 0.05.

To eliminate bias from single-animal sequencing datasets and clarify the disease specificity of hub genes in ALI, this study conducted further external validation using GEO public datasets. Receiver operating characteristic (ROC) curves were generated to evaluate the diagnostic efficacy of eight hub genes: *endothelin-1* (*EDN1*), *sphingosine kinase 1 (SPHK1)*, *intercellular adhesion molecule-1 (ICAM1)*, *RELA proto-oncogene encoded NF-κB p65 subunit (RELA)*, *WT1-associated protein (WTAP)*, *prostaglandin-endoperoxide synthase 2 (PTGS2/*COX2*)*, *interleukin-17A (IL17A)*, and *nuclear factor κB inhibitor alpha (NFKBIA)*. Single-gene analysis identified *EDN1* as having the optimal discriminatory power (AUC = 0.943). Figure 4A shows the five-gene combined diagnostic model consisting of *RELA*, *WTAP*, *EDN1*, *SPHK1*, and *ICAM1*, with an AUC of 0.961 (95% CI: 0.928–0.988). Figure 4B presents the seven-gene combined diagnostic model consisting of EDN1, SPHK1, ICAM1, RELA, PTGS2, IL17A, and WTAP, with an AUC of 0.966 (95% CI: 0.936–0.989). Figure 4C displays the eight-gene combined diagnostic model including all screened hub genes, with an AUC of 0.969 (95% CI: 0.940–0.991). Comprehensive comparison showed that the eight-gene combined model had the best disease discrimination value for ALI. Immune infiltration correlation heatmap analysis results are presented in Figure 4D. The associations between eight genes (*EDN1*, *ICAM1*, *IL17A*, *NFKBIA*, *PTGS2*, *RELA*, *SPHK1*, and *WTAP*) and the infiltration abundance of 28 immune cell types were evaluated. Overall, the expression levels of six genes (*RELA*, *SPHK1*, *IL17A*, *ICAM1*, *EDN1*, and *PTGS2*) showed significant or extremely significant positive correlations with the infiltration abundance of pro-inflammatory immune cells, including Th1 cells, Th17 cells, and neutrophils. Specifically, *EDN1* and *SPHK1* expression levels exhibited extremely significant negative correlations with the infiltration abundance of central memory CD8^+^ T cells. High expression of these inflammation-related genes drove massive infiltration of pro-inflammatory cells and simultaneously inhibited the recruitment of protective central memory CD8^+^ T cells, collectively shaping a pro-inflammatory-biased immune microenvironment. Subsequent detection of myeloperoxidase (MPO), a specific marker for neutrophils, validated this inflammatory infiltration feature.

### 3.5. Evaluation of Neutrophil Infiltration and Core Protein Expression in Rat Lung Tissues by Immunohistochemical Staining

**Figure 5:**
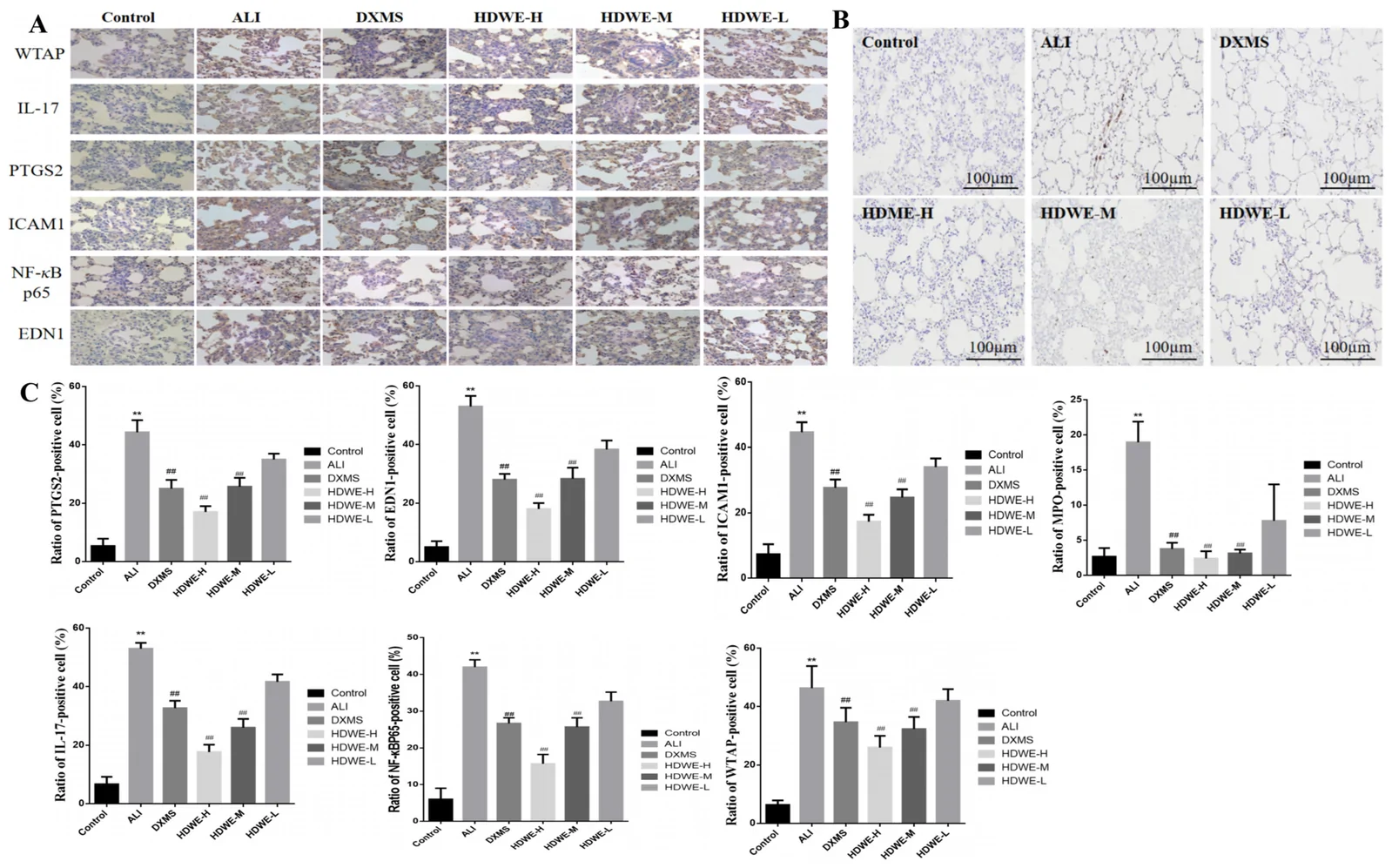
Immunohistochemical analysis of MPO and key functional proteins in lung tissues of rats in each group. (**A**) Representative immunohistochemical images of WTAP, IL-17, PTGS2 (COX-2), ICAM1, NF-κB p65, and EDN1 in the Control, ALI, DXMS, HDWE-H, HDWE-M, and HDWE-L groups. (**B**) Representative raw MPO immunohistochemical micrographs showing MPO-positive neutrophils (brown cytoplasm) and all cell nuclei stained blue by hematoxylin, which reflect neutrophil infiltration in lung tissues of each group. Scale bar = 100 μm. (**C**) Quantitative analysis of the positive-staining area ratio for each target protein and the MPO-positive cell ratio. Data are expressed as mean ± SD. ** *p* < 0.01 versus Control group. ^##^ *p* < 0.01 versus ALI group.

Immunohistochemical staining was performed to detect the expression levels of WTAP, IL-17, PTGS2/COX-2, ICAM1, NF-κB p65, and EDN1, and to evaluate MPO-positive neutrophil infiltration in rat lung tissues. Representative immunohistochemical images are presented in Figure 5A,B, and the corresponding quantitative analysis results are shown in Figure 5C. For MPO staining, representative segmented-rendered images are provided in Figure S1, and grouped quantitative data of the MPO-positive cell ratio are summarized in Table S6. Compared with the Control group, the ALI group showed markedly increased positive staining of inflammation-related proteins, accompanied by extensive MPO-positive neutrophil infiltration in the lung interstitium (*p* < 0.01). Intervention with DXMS and graded doses of HDWE significantly downregulated these abnormally increased protein expressions. Among them, DXMS, HDWE-H, and HDWE-M produced more prominent protective effects.

### 3.6. Chemical Composition Analysis of Hedyotis diffusa

**Figure 6:**
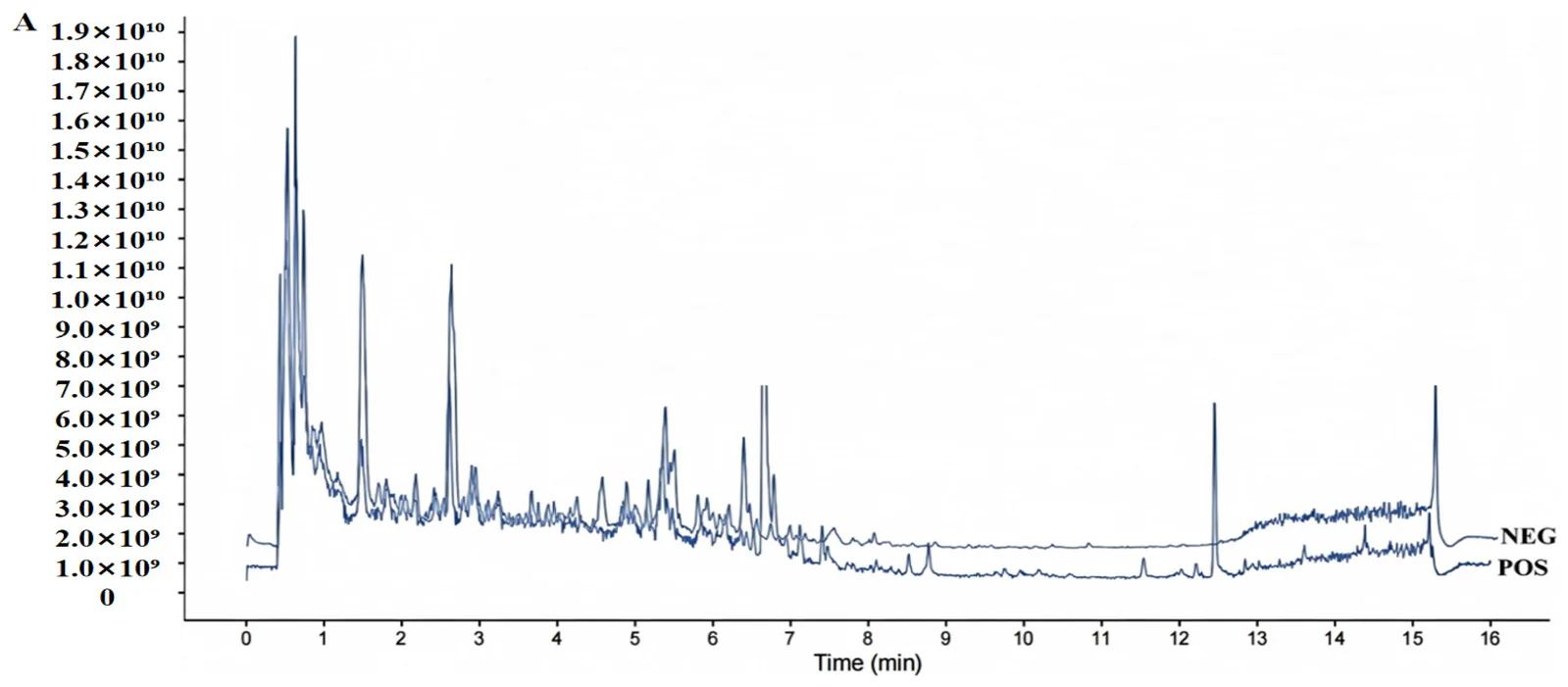

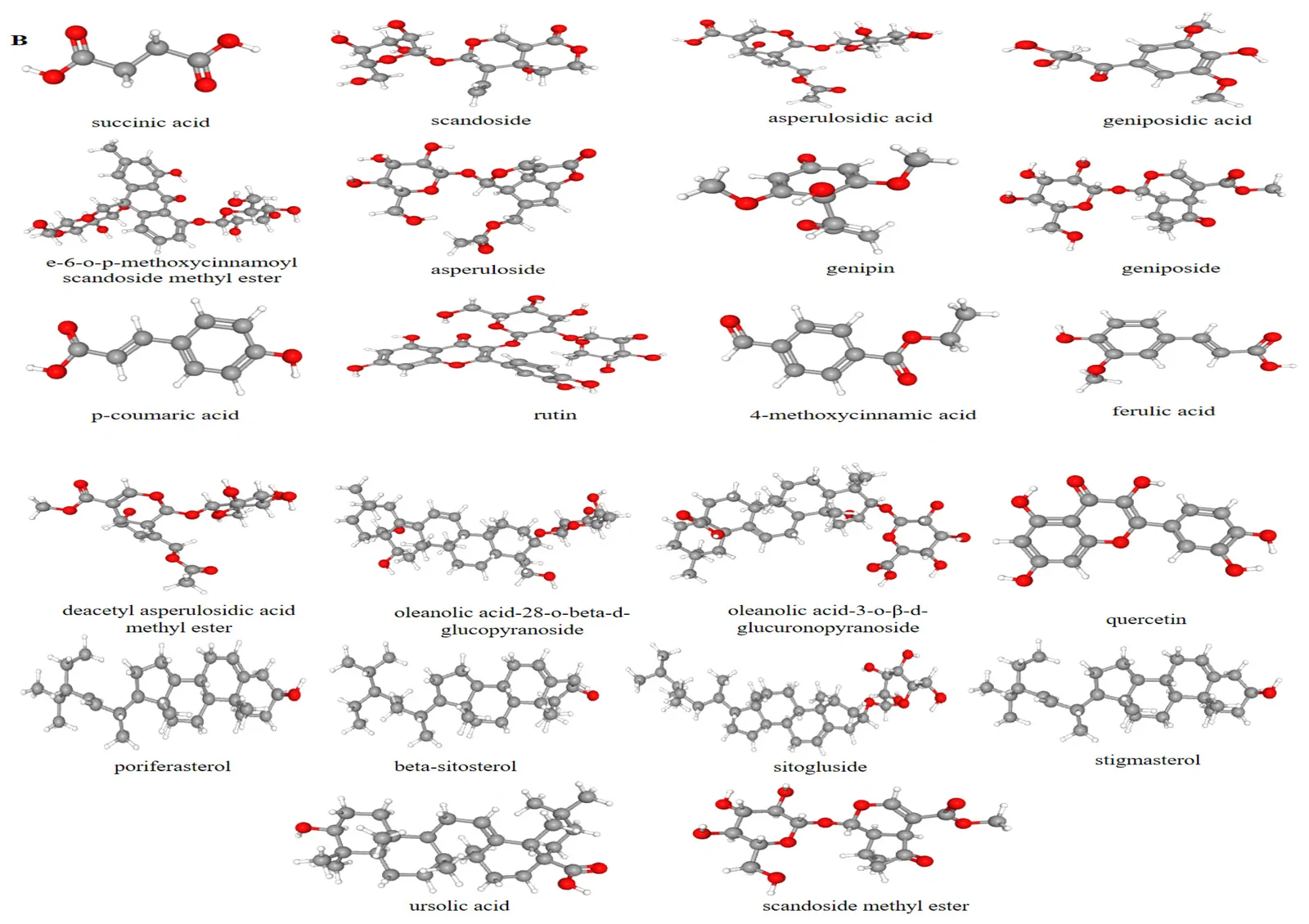
Representative chromatograms in positive and negative ion modes and the corresponding chemical structures. (**A**) Overlaid total ion chromatograms obtained under positive (POS) and negative (NEG) electrospray ionization modes. (**B**) Three-dimensional molecular structures of the major bioactive constituents in HDWE. Gray balls stand for carbon atoms, red balls for oxygen atoms, and white balls for hydrogen atoms. All 22 compounds are labeled below each corresponding structural formula.

UHPLC-Q-Orbitrap HRMS was used to characterize the chemical constituents of HDWE. Total ion chromatograms were acquired under positive and negative electrospray ionization modes, and three-dimensional molecular structures of the identified major bioactive compounds have been presented (Figure 6). Figure 6A presents the overlaid total ion chromatograms obtained under POS and NEG modes, while Figure 6B displays the three-dimensional molecular structures of the 22 major bioactive constituents in HDWE. UHPLC-Q-Orbitrap HRMS profiling detected 104 chemical constituents (Table S7), and screening using the TCMSP database identified 37 candidate bioactive compounds (Table S8). Intersection screening included structurally identical monomers and homologous derivatives sharing the same core skeleton but differing only in glycosyl, hydroxyl, or methyl ester substituents, resulting in the identification of 22 overlapping bioactive constituents (Table 1).

### 3.7. Molecular Docking Analysis and Ligand-Target Interactions

**Figure 7:**
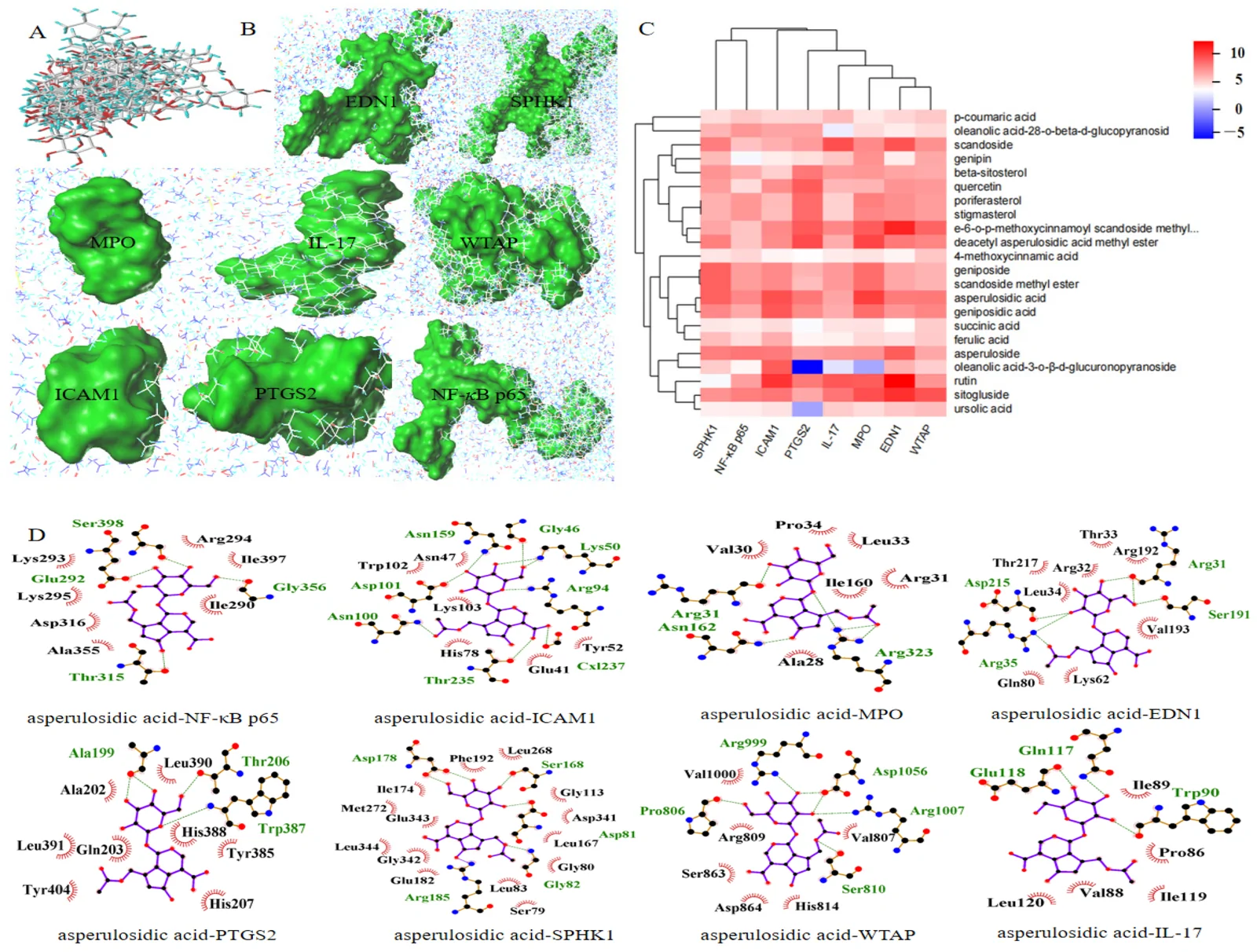
Molecular docking analysis. (**A**) Depicts the optimized rod-shaped model of the active pharmaceutical ingredient, comprising 22 ligand molecules, with red representing hydrogen atoms and blue representing oxygen atoms. (**B**) Three-dimensional binding pockets of the 8 target proteins. (**C**) Heatmap of docking binding energies between the compounds and their target proteins. The color bar represents docking scores: red indicates high docking scores reflecting strong binding capacity between molecules and target proteins, whereas blue indicates low docking scores reflecting weak binding capacity. (**D**) Two-dimensional ligand–receptor interaction diagrams of asperulosidic acid docked with NF-*κ*B p65, ICAM1, EDN1, MPO, PTGS2, SPHK1, WTAP and IL-17 proteins. Green dotted lines represent hydrogen bonds formed between asperulosidic acid and amino acid residues of target proteins, and the red curved lines indicate hydrophobic interaction residues around the binding pocket.

A docking score greater than 5 was used as the threshold for identifying favorable predicted ligand–protein interactions. In silico molecular docking was performed to evaluate the interactions of 22 candidate compounds with eight ALI-associated protein targets: EDN1, SPHK1, ICAM1, IL-17, PTGS2/COX2, NF-κB p65, WTAP, and MPO. Figure 7A shows the optimized structures of the candidate compounds, Figure 7B presents the binding pockets of the eight target proteins, and Figure 7C displays a heatmap of the predicted docking scores for the compound–target pairs. Sixteen compounds met the predefined docking threshold, and most showed favorable predicted interactions with multiple protein targets. Among these constituents, asperulosidic acid showed favorable predicted interactions with all eight ALI-associated protein targets. The two-dimensional interaction diagrams between asperulosidic acid and each target protein are shown in Figure 7D. Docking analysis predicted that asperulosidic acid formed hydrogen bonds with residues such as Asn, Glu, Thr, Arg, and Gly within the predicted binding pockets and hydrophobic contacts with residues including Leu, Val, Trp, and Phe. These interactions may contribute to stabilization of the ligand within the protein-binding pockets. Overall, the docking results suggest that asperulosidic acid may interact with multiple ALI-associated protein targets, providing a basis for subsequent molecular dynamics and functional validation.

### 3.8. Molecular Dynamics and Binding Free Energy Analysis of Selected Protein–Ligand Complexes

#### 3.8.1. Molecular Dynamics Simulations

**Figure 8:**
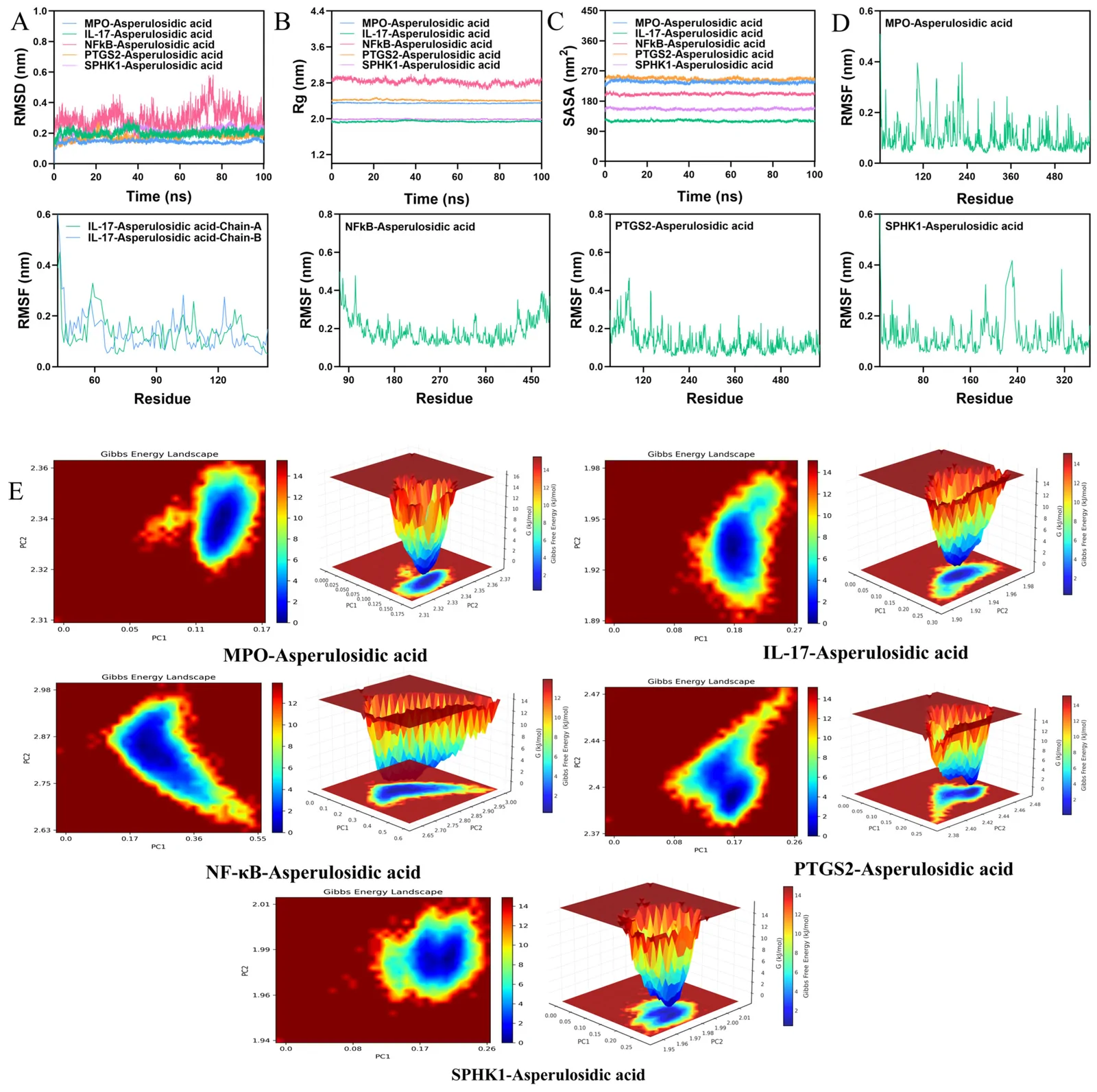
Molecular dynamics analysis of protein–asperulosidic acid complexes. (**A**) Root mean square deviation (RMSD) profiles of the MPO, IL-17, NF-κB p65, PTGS2/COX2, and SPHK1 complexes during the 100 ns molecular dynamics simulations. (**B**) Radius of gyration (Rg) profiles showing the overall compactness of the five protein–ligand complexes. (**C**) Solvent-accessible surface area (SASA) profiles of the five complexes during the simulations. (**D**) Root mean square fluctuation (RMSF) profiles of amino acid residues in the five protein–ligand complexes. (**E**) Gibbs free energy landscapes (FELs) of the five complexes, in which the blue regions represent low-free-energy conformational states.

Following molecular docking of the eight ALI-associated protein targets, five selected protein targets (MPO, IL-17, NF-κB p65, PTGS2/COX2, and SPHK1) were subjected to 100 ns molecular dynamics (MD) simulations to evaluate the dynamic stability of the asperulosidic acid–protein complexes. As shown in Figure 8A, the MPO–asperulosidic acid complex reached equilibrium at approximately 10 ns with an RMSD fluctuation of about 0.14 nm. The IL-17, NF-κB p65, PTGS2/COX2, and SPHK1 complexes stabilized at approximately 40, 90, 25, and 90 ns, with RMSD values of about 0.22, 0.24, 0.21, and 0.23 nm, respectively. Overall, the five complexes maintained relatively low RMSD fluctuations after equilibration. The Rg profiles remained relatively stable throughout the simulations, indicating no substantial changes in overall complex compactness (Figure 8B). SASA profiles also showed only minor fluctuations, suggesting relatively stable solvent exposure of the complexes (Figure 8C). Most residues in the MPO, NF-κB p65, and PTGS2/COX2 complexes exhibited RMSF values below 0.4 nm, whereas those in the IL-17 and SPHK1 complexes generally remained below 0.3 nm, indicating limited residue fluctuations (Figure 8D). The Gibbs free energy landscapes identified low-energy conformational states for all five complexes (Figure 8E). The minimum-energy coordinates were PC1 = 0.14 and PC2 = 2.335 for MPO, PC1 = 0.14 and PC2 = 1.945 for IL-17, PC1 = 0.25 and PC2 = 2.800 for NF-κB p65, PC1 = 0.18 and PC2 = 2.455 for PTGS2/COX2, and PC1 = 0.23 and PC2 = 1.975 for SPHK1.

#### 3.8.2. MM/PBSA Binding Free Energy Calculation of Protein–Ligand Complexes

**Figure 9:**
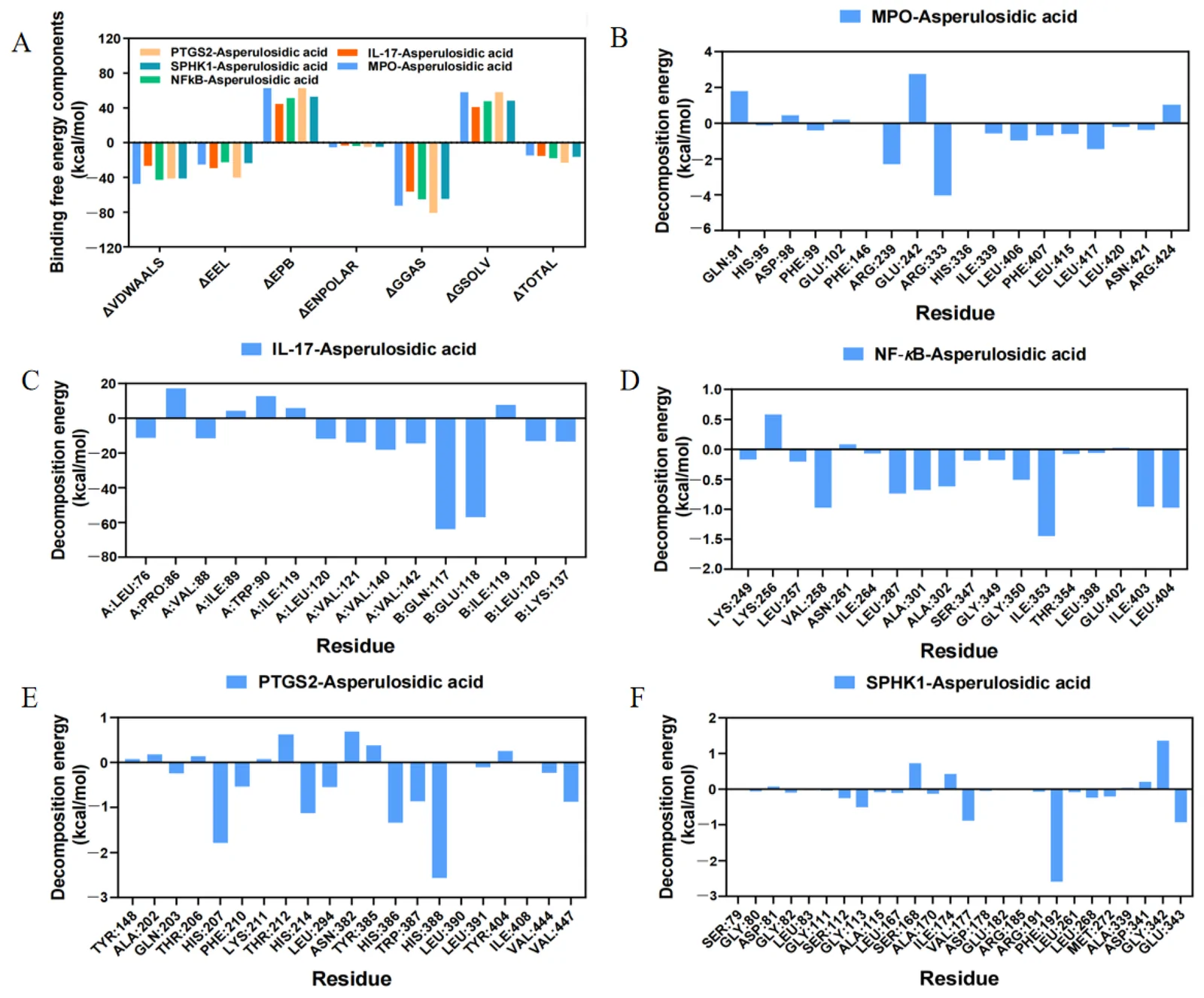
MM/PBSA binding free energy analysis of protein–ligand complexes. (**A**) Calculated binding free energies of the MPO, IL-17, NF-κB p65, PTGS2/COX2, and SPHK1 complexes with asperulosidic acid. (**B**–**F**) Residue-wise energy decomposition analyses of the MPO, IL-17, NF-κB p65, PTGS2/COX2, and SPHK1 complexes, respectively.

On the basis of stable conformations obtained from molecular dynamics simulations, the Molecular Mechanics/Poisson–Boltzmann Surface Area (MM/PBSA) method quantifies binding free energies between asperulosidic acid and core target proteins (Figure 9A). Calculated binding free energies for complexes of asperulosidic acid with MPO, IL-17, NF-κB p65, PTGS2 (COX2), and SPHK1 are −14.74, −14.92, −17.58, −23.04, and −16.1 kcal/mol, respectively. Negative values across all systems demonstrate favorable binding affinity of the small molecule toward each target protein. Energy decomposition analysis identifies critical amino acid residues contributing to binding within each complex (Figure 9B–F). Key residues include ARG333, ARG239, LEU417, LEU406, PHE407, LEU415, and ILE339 for MPO; B: GLN117, B: GLU118, A: VAL140, A: VAL142, A: VAL121, B: LYS137, B: LEU120, A: LEU120, A: ALA188, and A: LEU176 for IL-17; ILE353, VAL258, LEU404, ILE403, LEU287, ALA301, ALA302, and GLY350 for NF-κB p65; HIS388, HIS207, HIS386, HIS214, TRP387, VAL447, LEU294, and PHE210 for PTGS2; and PHE192, GLU343, VAL177, and GLY113 for SPHK1 These specific residues dominate small-molecule recognition, anchoring, and stable binding to target proteins, further verifying stable and specific interactions between asperulosidic acid and the five core targets associated with acute lung injury.

### 3.9. RT-qPCR Analysis of SPHK1, RELA, and NFKBIA mRNA Expression in Lung Tissues

**Figure 10:**
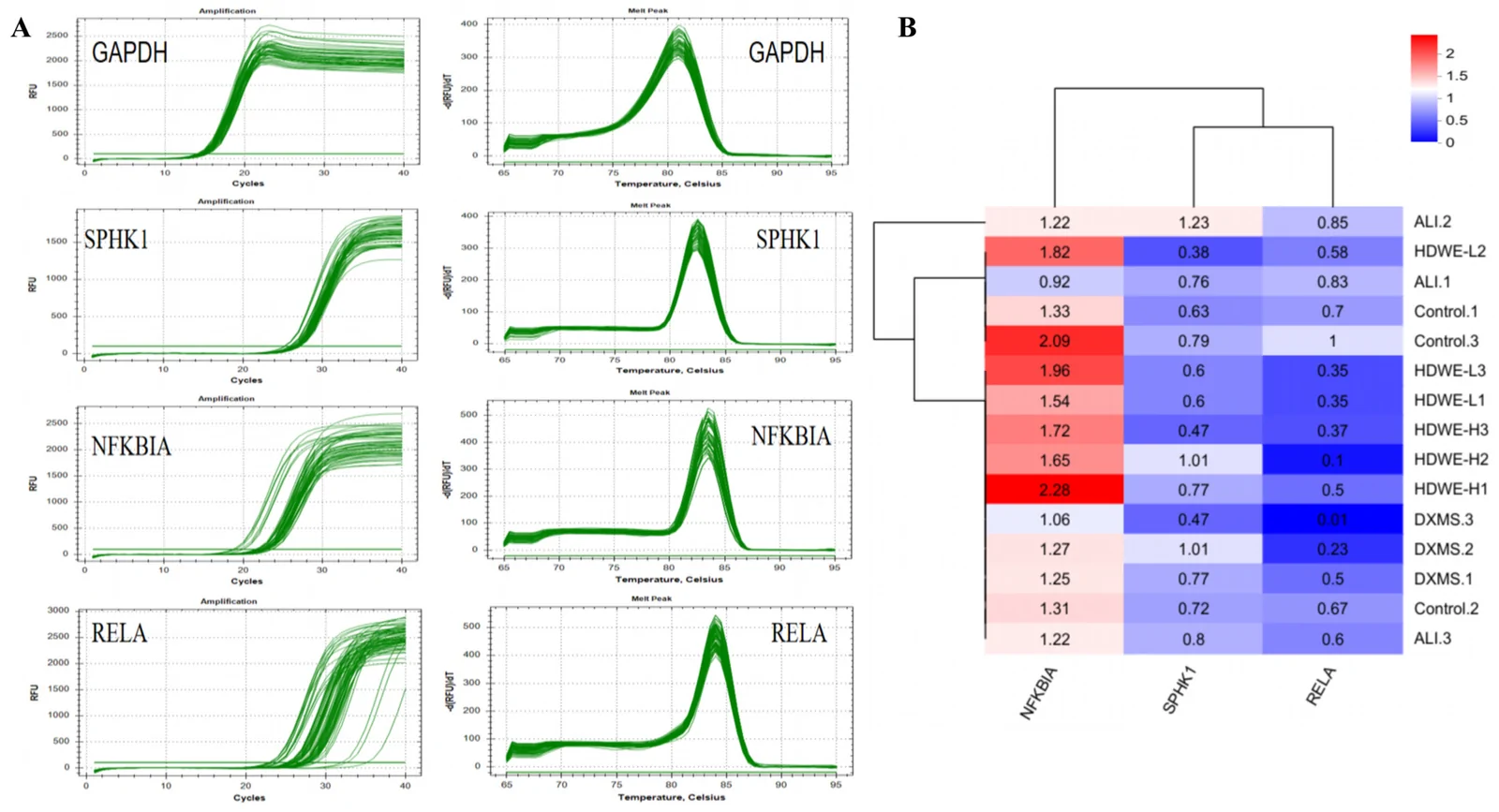
RT-qPCR analysis of *SPHK1*, *RELA*, and *NFKBIA* mRNA expression in rat lung tissues. (**A**) Amplification and melting curves of the target genes and the reference gene *GAPDH*, showing the specificity of primer amplification. (**B**) Heatmap illustrating the relative mRNA expression patterns of *SPHK1*, *RELA*, and *NFKBIA* across the experimental groups.

RT-qPCR was performed to evaluate the relative mRNA expression levels of *SPHK1*, *RELA*, and *NFKBIA* in lung tissues. Figure 10A shows the amplification and melting curves of the target genes and the reference gene *GAPDH*, indicating the specificity of primer amplification. Figure 10B presents a heatmap showing the relative expression patterns of *SPHK1*, *RELA*, and *NFKBIA* among the experimental groups. Compared with the Control group, *SPHK1* expression in the ALI group increased to approximately 1.30-fold, whereas *RELA* expression remained close to the Control group level (0.96-fold). The mRNA expression level of *NFKBIA* in the ALI group decreased to 0.71-fold compared with the Control group. Compared with the ALI group, HDWE-H and HDWE-L treatment reduced *SPHK1* expression to 0.62-fold and 0.57-fold, respectively. *RELA* expression was reduced to 0.33-fold in the DXMS group and 0.43-fold in the HDWE-H group (*p* < 0.05), while HDWE-L treatment reduced *RELA* expression to 0.56-fold. In addition, HDWE-H and HDWE-L treatments increased *NFKBIA* expression to 1.68-fold and 1.58-fold, respectively, compared with the ALI group (*p* < 0.05). The relative expression levels of these genes are provided in Table S9.

## 4. Discussion

**Figure 11:**
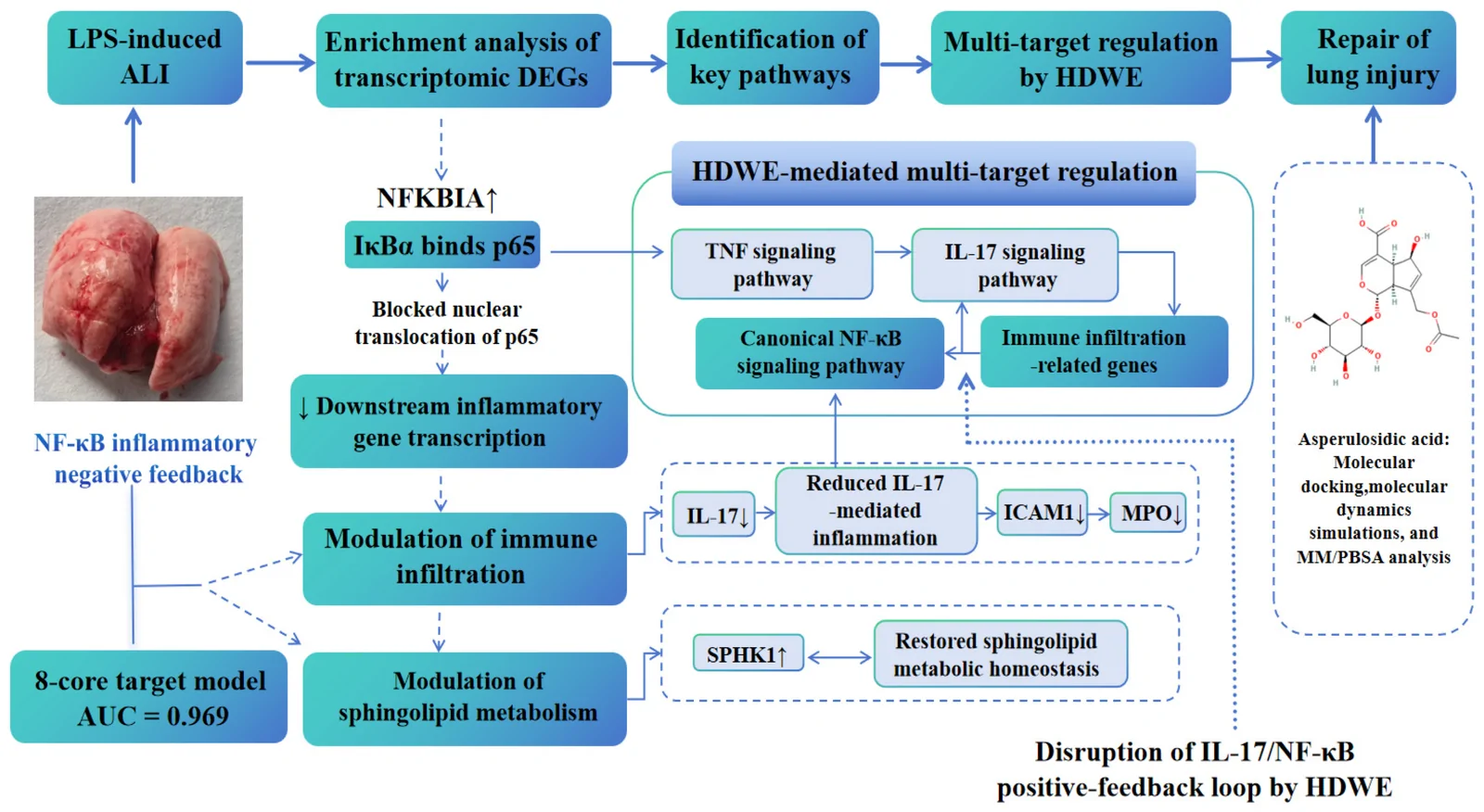
Schematic overview of the potential multi-target mechanisms of HDWE in LPS-induced ALI. HDWE treatment was associated with increased *NFKBIA* expression, reduced *SPHK1* expression, and decreased levels of IL-17, ICAM1, MPO, and NF-κB p65, suggesting potential modulation of the IL-17/NF-κB inflammatory axis and inflammatory cell infiltration. Asperulosidic acid showed predicted interactions with eight ALI-associated protein targets based on molecular docking and simulation analyses and may represent a potential active constituent of HDWE. The diagnostic model constructed using eight hub genes achieved an AUC of 0.969 for ALI discrimination. Solid arrows represent experimentally validated regulatory cascades in this study. Dashed arrows denote inferred associations from transcriptomic analysis or previously reported feedback loops.

Previous pharmacological studies have suggested that iridoid glycosides possess anti-inflammatory, antioxidant, and pulmonary protective properties. These compounds may alleviate LPS-induced lung injury through mechanisms involving the attenuation of inflammatory infiltration, oxidative stress regulation, and modulation of the NF-κB signaling pathway [30,31]. Asperulosidic acid, a major iridoid constituent of *Hedyotis diffusa*, has been reported to regulate NF-κB and MAPK signaling pathways, reduce the production of pro-inflammatory mediators, including TNF-α, IL-6, and PGE_2_, in macrophages, and interact with NF-κB p65. These reported effects are consistent with the target enrichment results obtained in the present study. Previous in vitro studies have suggested that asperulosidic acid and asperuloside derived from *Hedyotis diffusa* reduce PTGS2 expression in macrophages and suppress RELA nuclear translocation, thereby decreasing inflammatory cytokine production [32]. In addition, asperulosidic acid has been reported to downregulate *ICAM1* expression and reduce inflammatory cell adhesion and endothelial transmigration [33]. However, the regulatory effects of these iridoid glycosides on *WTAP*, *SPHK1*, *EDN1*, and MPO-related inflammatory responses remain insufficiently characterized. In the present study, molecular docking, molecular dynamics simulations, and MM/PBSA binding free energy calculations suggested that multiple iridoid glycosides in HDWE exhibited favorable interactions with eight ALI-associated protein targets. Among these compounds, asperulosidic acid showed potential multi-target interaction properties, while related compounds, including scandoside, deacetyl asperulosidic acid methyl ester, asperuloside, and geniposidic acid, also exhibited potential interactions with multiple targets. These findings suggest that these constituents may contribute to the pharmacological effects of HDWE. Furthermore, molecular dynamics simulations indicated that asperulosidic acid formed relatively stable complexes with MPO, IL-17, NF-κB p65, PTGS2/COX2, and SPHK1 based on conformational stability and binding free energy analyses. However, these interactions were predicted by computational approaches, and further in vivo and in vitro functional studies will be required to validate their biological relevance. Taken together, the multi-target pharmacological mechanisms of HDWE protecting against LPS-induced ALI are schematically illustrated in Figure 11, including disruption of the IL-17/NF-κB positive-feedback loop, regulation of immune infiltration and sphingolipid metabolism, the diagnostic performance of the model constructed using eight hub genes, and computational interaction predictions for iridoid glycoside constituents.

ALI is characterized by excessive inflammatory activation, disruption of the alveolar microvascular barrier, and early alterations in the pulmonary interstitial matrix [34]. In the present study, LPS stimulation disrupted lung tissue architecture in rats, resulting in alveolar wall thickening, widening of the alveolar septa, and extensive inflammatory cell infiltration, whereas HDWE treatment partially alleviated these pathological changes. Masson’s trichrome staining further showed that HDWE reduced abnormal collagen deposition in lung tissues, suggesting a potential effect on early extracellular matrix remodeling during ALI progression. LPS-induced inflammatory activation is associated with increased levels of TNF-α, IL-1β, and IL-6 and decreased IL-10, which may aggravate pulmonary inflammatory injury [35,36,37]. In this study, HDWE treatment modulated these inflammatory cytokine levels, reduced MPO-positive neutrophil infiltration, and downregulated ICAM1 expression. These findings suggest that HDWE may attenuate local inflammatory injury, potentially in part through effects on inflammatory cell adhesion and migration.

Transcriptomic enrichment analysis showed that the DEGs identified after HDWE intervention were mainly enriched in the TNF, IL-17, and NF-κB signaling pathways. Immune infiltration correlation analysis indicated that the expression patterns of *RELA*, *SPHK1*, and *IL17A* were associated with the infiltration of Th1 cells, Th17 cells, and neutrophils in lung tissues, suggesting that HDWE may influence both innate and adaptive immune responses during ALI progression. Immunohistochemical analysis showed that HDWE treatment reduced the elevated expression of WTAP, IL-17, PTGS2/COX2, ICAM1, NF-κB p65, EDN1, and MPO in lung tissues. These changes may be associated with attenuation of EDN1-related endothelial injury and inflammatory responses. In addition, the observed changes in IL-17, NF-κB p65, PTGS2/COX2, and ICAM1 suggest that HDWE may modulate the IL-17/NF-κB inflammatory axis and thereby reduce inflammatory cell infiltration. The reduction in WTAP expression may also be associated with attenuation of inflammatory signaling, although its specific role requires further investigation. RT-qPCR analysis showed that *SPHK1* expression was increased in the ALI group, whereas HDWE treatment partially attenuated this increase, suggesting that regulation of *SPHK1* may be involved in the effects of HDWE on sphingolipid-associated inflammatory responses. *NFKBIA* encodes IκBα, which binds to NF-κB p65 and inhibits its nuclear translocation, thereby acting as an important negative regulator of NF-κB signaling [38]. In this study, HDWE increased *NFKBIA* expression and slightly decreased *RELA* expression, suggesting that modulation of these genes may contribute to the attenuation of excessive NF-κB signaling. Furthermore, the diagnostic model constructed using eight hub genes achieved an AUC of 0.969 for ALI discrimination, suggesting that this gene combination may have potential value as a biomarker panel for ALI.

This study has several limitations. First, the mechanistic interpretation was mainly based on transcriptomic analysis, molecular assays, and computational simulations. Target inhibition, gene overexpression, and gene knockout experiments were not performed; therefore, a direct causal relationship between the identified targets and the pulmonary protective effects of HDWE could not be established. The current mechanistic evidence should therefore be considered as indirect evidence requiring further validation. Second, the in vivo experiments were limited to an LPS-induced rat model of ALI and did not include other clinically relevant causes of lung injury, such as viral infection or mechanical ventilation. Therefore, whether HDWE exhibits differential effects in ALI induced by different etiological factors remains unclear. In addition, direct pulmonary function parameters, including PaO_2_/FiO_2_ and lung compliance, were not evaluated. The relatively limited sample size of transcriptomic sequencing may also affect the robustness of the bioinformatic analysis. At the protein level, immunohistochemical analysis was performed for validation, whereas quantitative confirmation using methods such as Western blotting was not conducted. Among the transcriptomic findings, *ELANE* did not show significant differential expression and was not included in the subsequent validation experiments. Its potential regulatory role will require further investigation. Furthermore, the present study did not include comparisons with public ALI transcriptomic datasets, and the broader applicability of the proposed molecular mechanisms remains to be determined. The interactions between individual iridoid glycosides and ALI-associated protein targets were predicted by computational approaches, and further cellular and animal studies using isolated compounds will be needed to validate their pharmacological effects.

Future studies will focus on further validating the roles of key targets, particularly SPHK1 and NFKBIA, using pharmacological inhibition and gene-editing approaches. Individual iridoid glycosides will be isolated and purified, and their pharmacological effects will be evaluated in vitro and in vivo. Additional approaches, including ECIS/TER assays, will be applied to assess alveolar barrier function. Moreover, ALI models induced by different stimuli will be established to compare the effects of HDWE across different injury conditions, and multi-omics analyses will be integrated to further characterize the molecular regulatory network underlying HDWE treatment.

## 5. Conclusions

This study investigated the protective effects and potential mechanisms of *Hedyotis diffusa* water extract (HDWE) against LPS-induced acute lung injury in rats using pharmacodynamic evaluation, transcriptomic analysis, liquid chromatography–mass spectrometry, and molecular simulations. HDWE alleviated lung tissue injury, collagen deposition, and neutrophil infiltration and modulated inflammatory cytokine levels. Transcriptomic analysis showed that the DEGs were mainly enriched in the NF-κB, IL-17, and TNF signaling pathways. HDWE increased *NFKBIA* expression and reduced *SPHK1* expression, suggesting that modulation of IL-17/NF-κB-related inflammatory responses may contribute to its protective effects. The diagnostic model based on eight hub genes showed high discriminatory performance for ALI (AUC = 0.969). Twenty-two major constituents of HDWE were identified, and molecular docking, molecular dynamics simulations, and MM/PBSA binding free energy calculations suggested that asperulosidic acid exhibited favorable interactions with multiple ALI-associated protein targets and may represent an important active constituent of HDWE. The present study is limited by the lack of genetic functional validation, in vivo ADME evaluation of individual compounds, and the use of a single LPS-induced ALI model. Nevertheless, these findings provide a basis for further investigation of the active constituents and molecular mechanisms of HDWE. Future studies will further validate key targets and evaluate the pharmacological effects of individual active compounds.

## Figures and Tables

**Table 1:** 22 chemical constituents identified from HDWE.

NO.	RT (min)	Name	Formula	Fragment Ion
1	0.59	succinic acid	C_4_H_6_O_4_	117.0190; 73.0292; 99.0083
2	0.66	scandoside	C_16_H_22_O_10_	373.1138; 211.0603; 149.0602
3	0.75	asperulosidic acid	C_18_H_24_O_12_	431.1179; 165.0551; 59.0139; 89.0241
4	0.82	geniposidic acid	C_11_H_14_O_6_	241.0714; 197.0456; 169.0507
5	0.83	e-6-o-p-methoxycinnamoyl scandoside methyl ester	C_27_H_32_O_13_	563.1768; 413.1202; 179.0702
6	0.88	asperuloside	C_18_H_22_O_11_	437.1015; 275.0541; 147.0443; 197.0212
7	0.92	genipin	C_11_H_14_O_5_	227.0912; 209.0807; 181.0855
8	1.00	geniposide	C_17_H_24_O_10_	411.1221; 339.0905; 217.0462; 249.0734
9	1.48	p-coumaric acid	C_9_H_8_O_3_	147.0442; 119.0491; 91.0538
10	1.53	rutin	C_27_H_30_O_16_	609.1469; 300.0281; 301.0360; 271.0257
11	1.82	4-methoxycinnamic acid	C_10_H_10_O_3_	179.0702; 161.0588; 133.0641
12	1.82	ferulic acid	C_10_H_10_O_4_	193.0493; 134.0364; 178.0277
13	2.61	deacetyl asperulosidic acid methyl ester	C_19_H_26_O_12_	387.1271; 207.0654; 147.0443; 175.0379
14	2.94	oleanolic acid-28-o-beta-d-glucopyranoside	C_36_H_58_O_8_	613.4056; 455.3527; 393.2904
15	2.96	oleanolic acid-3-o-β-d-glucuronopyranoside	C_36_H_56_O_10_	639.3798; 455.3526; 191.0551
16	3.46	quercetin	C_15_H_10_O_7_	303.0489; 153.0179; 229.0494; 137.0227
17	5.44	poriferasterol	C_29_H_48_O	413.3775; 147.1001; 161.1164
18	6.46	beta-sitosterol	C_29_H_50_O	415.3931; 149.1158; 163.1322
19	9.21	sitogluside	C_35_H_60_O_6_	577.4515; 415.3934; 163.1321
20	12.06	stigmasterol	C_29_H_48_O	395.3664; 145.1003; 159.1166; 81.0694
21	12.53	ursolic acid	C_30_H_48_O_3_	439.3566; 203.1790; 191.1793; 95.0857
22	12.73	scandoside methyl ester	C_17_H_24_O_10_	411.1223; 217.0462; 149.0601

## Data Availability

The public microarray datasets analyzed were available from the NCBI GEO database under accession numbers GSE3077, GSE40885, and GSE5272 (https://www.ncbi.nlm.nih.gov/geo/ (accessed on 10 June 2025)). The raw RNA-seq data generated in this study are publicly available in the Genome Sequence Archive (GSA) accession CRA047963 (https://ngdc.cncb.ac.cn/gsa/search?searchTerm=CRA047963) (accessed on 9 August 2026). Additional data supporting the findings of this study are provided within the article and its Supplementary Materials.

## Supplementary Materials

The following supporting information can be downloaded at https://www.mdpi.com/article/10.3390/biology15171549/s1. Table S1: Chromatographic gradient elution program; Table S2: Primers used for RT-qPCR; Table S3: Pathway analysis involving differential genes; Table S4: Pathways associated with acute lung injury; Table S5: Key KEGG pathways associated with ALI and corresponding validation molecules; Table S6: Composition determined by UHPLC-MS/MS; Table S7: TCMSP screening of chemical constituents in *Hedyotis diffusa*; Table S8: Relative expression levels of the relevant genes; Table S9: Relative expression levels of the relevant genes (2^−ΔΔCt^, mean ± SD).

## Abbreviations

The following abbreviations are used in this manuscript:

EDN1	Endothelin 1
WTAP	WT1-associated protein
IL-17	Interleukin-17
COX2	Cyclooxygenase-2
ICAM1	Intercellular adhesion molecule-1
NF-κB p65	Nuclear factor-kappa-B p65
DAB	Diaminobenzidine
MPO	Myeloperoxidase
qPCR	Real-Time Quantitative Polymerase Chain Reaction
ROC	Receiver Operating Characteristic
PME	Particle-Mesh Ewald
RMSF	Root Mean Square Fluctuation
GAPDH	Glyceraldehyde-3-phosphate dehydrogenase
PCA	Principal Component Analysis
GO	Gene Ontology
KEGG	Kyoto Encyclopedia of Genes and Genomes
