# Supplementary material for: Mechanism of Action of Hedyotis diffusa Extract in a Rat Model of Acute Lung Injury Based on Transcriptomic Analysis
Source: Biology (Basel). 2026 Sep 4;15(17):1549. doi: 10.3390/biology15171549 (PMC13564518; doi:10.3390/biology15171549)
Supplement: Supplementary file 1 [file biology-15-01549-s001.zip › biology-4494672-supplementary.pdf]

Supplements: Mechanism of Action of *Hedyotis diffusa* Extract in a Rat Model of Acute Lung Injury Based on Transcriptomic Analysis

Chenyi Lu , Xinyi Yang and Xin Yang *

Figure S1. Representative immunohistochemical images of MPO for the assessment of neutrophil infiltration in lung tissues of rats from each group. Brown-yellow staining indicates MPO-positive neutrophils. The purple overlay corresponds to the cell-segmentation rendering generated by Aipathwell® digital-pathology analysis software. The visible dividing lines represent intrinsic analytical boundaries derived from the built-in cell-segmentation algorithm of the software, rather than artifacts caused by image splicing. The MPO-positive cell ratio was calculated using the following formula: MPO-positive cell ratio (%) = (number of MPO-positive cells / total number of cells) × 100%. Scale bar = 100 μm.

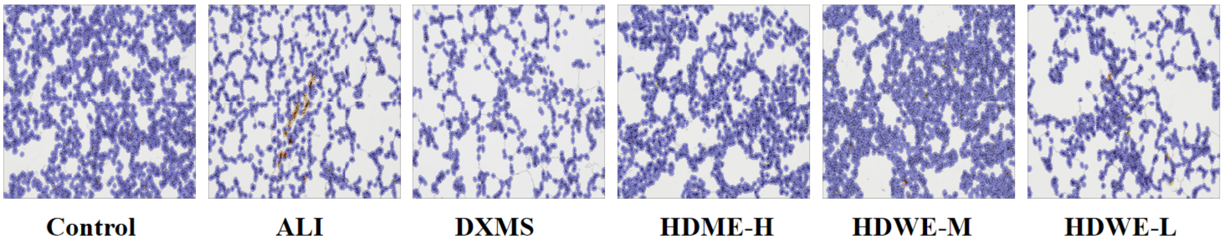

Table S1. Chromatographic gradient elution program.

Time (min)	Flow rate (μL/min)	A% water	B% acetonitrile
0	500	85	15
11	500	25	75
12	500	2	98
14	500	2	98
14.1	500	85	15
15	500	85	15
16	500	85	15

Table S2. Primers used for RT-qPCR.

Gene	Primer	Sequence (5′–3′)	Product length (bp)	Tm (℃)
GAPDH	Forward	CTGGAGAAACCTGCCAAGTATG	138	60
GAPDH	Reverse	GGTGGAAGAATGGGAGTTGCT	138	60
SPHK1	Forward	ACGAGGTGGTGAATGGGCTAA	128	60
SPHK1	Reverse	CTCGTGCCCAGCATAGTAGTTC	128	60

Gene	Primer	Sequence (5′–3′)	Product length (bp)	Tm (℃)
RELA	Forward	AACGCAAAAGGACCTACGAGA	156	60
RELA	Reverse	TTGAAAAGGCATAGGGCTGG	156	60
NFKBIA	Forward	TCGTGGAGCACTTGGTGACTT	161	60
NFKBIA	Reverse	GTAGCCCTGGTAGGTTACTCTGTTG	161	60

Table S3. Pathway analysis involving differential genes.

ID	Description	pvalue	p.adjust	qvalue
hsa04668	TNF signaling pathway	5.36E-18	8.52E-16	5.08E-16
hsa04657	IL-17 signaling pathway	3.90E-13	3.10E-11	1.85E-11
hsa04061	Viral protein interaction with cytokine and cytokine receptor	2.19E-11	1.07E-09	6.40E-10
hsa05164	Influenza A	3.11E-11	1.07E-09	6.40E-10
hsa04064	NF-kappa B signaling pathway	3.38E-11	1.07E-09	6.40E-10
hsa04060	Cytokine-cytokine receptor interaction	2.60E-10	6.88E-09	4.10E-09
hsa05323	Rheumatoid arthritis	5.44E-09	1.24E-07	7.36E-08
hsa04933	AGE-RAGE signaling pathway in diabetic complications	1.86E-07	3.67E-06	2.19E-06
hsa04621	NOD-like receptor signaling pathway	2.08E-07	3.67E-06	2.19E-06
hsa04625	C-type lectin receptor signaling pathway	2.52E-07	4.01E-06	2.39E-06
hsa04062	Chemokine signaling pathway	2.79E-07	4.04E-06	2.41E-06
hsa05144	Malaria	6.36E-07	8.43E-06	5.02E-06
hsa05417	Lipid and atherosclerosis	7.96E-07	9.74E-06	5.80E-06
hsa05134	Legionellosis	1.26E-06	1.43E-05	8.55E-06
hsa05171	Coronavirus disease - COVID-19	1.60E-06	1.69E-05	1.01E-05
hsa05162	Measles	2.20E-06	2.19E-05	1.30E-05
hsa05321	Inflammatory bowel disease	3.07E-06	2.73E-05	1.63E-05
hsa05143	African trypanosomiasis	3.21E-06	2.73E-05	1.63E-05
hsa05142	Chagas disease	3.27E-06	2.73E-05	1.63E-05
hsa05169	Epstein-Barr virus infection	4.30E-06	3.42E-05	2.04E-05
hsa04620	Toll-like receptor signaling pathway	4.79E-06	3.63E-05	2.16E-05
hsa04622	RIG-I-like receptor signaling pathway	5.18E-06	3.74E-05	2.23E-05
hsa05160	Hepatitis C	6.05E-06	4.18E-05	2.49E-05
hsa04217	Necroptosis	6.34E-06	4.20E-05	2.50E-05
hsa05133	Pertussis	7.71E-06	4.91E-05	2.92E-05
hsa05140	Leishmaniasis	8.33E-06	5.09E-05	3.03E-05
hsa04210	Apoptosis	2.19E-05	0.000128758	7.67E-05
hsa05222	Small cell lung cancer	2.33E-05	0.000132189	7.88E-05
hsa05418	Fluid shear stress and atherosclerosis	2.52E-05	0.000138105	8.23E-05
hsa01523	Antifolate resistance	3.53E-05	0.000187191	0.000111534
hsa05146	Amoebiasis	4.19E-05	0.000214766	0.000127964
hsa05163	Human cytomegalovirus infection	7.80E-05	0.000387327	0.000230781
hsa05152	Tuberculosis	0.000130466	0.000628611	0.000374545
hsa05332	Graft-versus-host disease	0.000136327	0.00063753	0.000379859
hsa05132	Salmonella infection	0.000157913	0.000717378	0.000427435

hsa04623	Cytosolic DNA-sensing pathway	0.000171186	0.00075607	0.000450488
hsa04380	Osteoclast differentiation	0.000198968	0.000855026	0.00050945
hsa05167	Kaposi sarcoma-associated herpesvirus infection	0.000207621	0.000868729	0.000517614
hsa05135	Yersinia infection	0.00021558	0.000878902	0.000523675
hsa04936	Alcoholic liver disease	0.000261928	0.001041164	0.000620356
hsa04932	Non-alcoholic fatty liver disease	0.000419717	0.001627683	0.000969821
hsa04148	Efferocytosis	0.00043439	0.001644477	0.000979828
hsa05161	Hepatitis B	0.000531066	0.001963708	0.001170035
hsa04659	Th17 cell differentiation	0.00058282	0.002106098	0.001254875
hsa05145	Toxoplasmosis	0.000660529	0.00233387	0.001390588
hsa05202	Transcriptional misregulation in cancer	0.001326264	0.004584259	0.002731435
hsa05130	Pathogenic Escherichia coli infection	0.001512194	0.005115719	0.003048095
hsa04940	Type I diabetes mellitus	0.002500421	0.008282645	0.004935041
hsa05166	Human T-cell leukemia virus 1 infection	0.002696448	0.008749697	0.005213325
hsa04010	MAPK signaling pathway	0.002772039	0.008815083	0.005252284
hsa04640	Hematopoietic cell lineage	0.003509279	0.010940693	0.006518784
hsa04931	Insulin resistance	0.004790256	0.014647128	0.008727186
hsa04370	VEGF signaling pathway	0.006132193	0.01839658	0.010961219
hsa04650	Natural killer cell mediated cytotoxicity	0.009657299	0.027915583	0.01663292
hsa04920	Adipocytokine signaling pathway	0.009831904	0.027915583	0.01663292
hsa05120	Epithelial cell signaling in Helicobacter pylori infection	0.009831904	0.027915583	0.01663292
hsa05010	Alzheimer disease	0.010325644	0.028803111	0.017161734
hsa05170	Human immunodeficiency virus 1 infection	0.010817318	0.029654371	0.01766894
hsa04662	B cell receptor signaling pathway	0.016082441	0.043340815	0.025823723
hsa05165	Human papillomavirus infection	0.017880395	0.047383048	0.028232203
hsa05235	PD-L1 expression and PD-1 checkpoint pathway in cancer	0.018747881	0.048867427	0.02911664
hsa04666	Fc gamma R-mediated phagocytosis	0.023494127	0.059294701	0.035329514
hsa05410	Hypertrophic cardiomyopathy	0.023494127	0.059294701	0.035329514
hsa04066	HIF-1 signaling pathway	0.03172741	0.078822784	0.046964916

Table S4. Pathways associated with acute lung injury.

ID	Description	P-value	p.adjust	qvalue
hsa04668	TNF signaling pathway	5.36E-18	8.52E-16	5.08E-16
hsa04657	IL-17 signaling pathway	3.90E-13	3.10E-11	1.85E-11
hsa04064	NF-κB signaling pathway	3.38E-11	1.07E-09	6.40E-10
hsa04933	AGE-RAGE signaling pathway in diabetic complications	1.86E-07	3.67E-06	2.19E-06
hsa04621	NOD-like receptor signaling pathway	2.08E-07	3.67E-06	2.19E-06
hsa04062	Chemokine signaling pathway	2.79E-07	4.04E-06	2.41E-06
hsa05418	Fluid shear stress and atherosclerosis	2.52E-05	0.000138105	8.23E-05
hsa04148	Efferocytosis	0.00043439	0.001644477	0.000979828

ID	Description	P-value	p.adjust	qvalue
hsa04370	VEGF signaling pathway	0.006132193	0.01839658	0.010961219

Table S5. Key KEGG pathways associated with ALI and corresponding validation molecules.

Pathway	Key genes	Biological relevance
TNF signaling pathway	RELA, EDN1, ICAM1, COX2	Serves as an early initiating signal in LPS-induced ALI and activates the NF-κB pathway.
IL-17 signaling pathway	IL-17, RELA, COX2	Sustains the amplification of NF-κB-mediated pulmonary inflammatory exudation.
NF-kappa B signaling pathway	RELA, EDN1, ICAM1, COX2, SPHK1	Acts as a core transcriptional hub in ALI-associated inflammation. Nuclear translocation of RELA simultaneously regulates endothelial leakage, sphingolipid metabolism, and the synthesis of pro-inflammatory cytokines.
Cytokine-cytokine receptor interaction	IL-17, EDN1, ICAM1	Forms an upstream inflammatory factor interaction network that triggers downstream TNF and IL-17 signaling cascades, thereby amplifying the pulmonary inflammatory microenvironment.

Table S6. MPO-Positive cell ratio in rat lung tissues.

Group	MPO-Positive cells ratio (%)
Control	2.66 ±1.2
ALI	18.95±3.0**
DXMS	3.76±0.9 ##
HDWE-H	2.40±1.0 ##
HDWE-M	3.12±0.6 ##
HDWE-L	7.75±5.2 ##

MPO-positive neutrophils and total cells within each microscopic field were automatically counted by Aipathwell® digital-pathology analysis software. Detailed calculation of this index is described in the Materials and Methods section. Data are expressed as mean ± SD.***P* < 0.01 vs control group； ##*P* < 0.01 vs model group.

Table S7. Composition determined by UHPLC-Q-Orbitrap HRMS.

NO.	English Name of the Compound	NO.	English Name of the Compound
1	Kaempferol	53	Calycosin
2	Stigmasterol	54	Daidzein
3	Geniposide	55	Naringenin
4	10-O-P-Hydroxybenzoyl Scandoside Methyl Ester	56	Quercetin-3-O-Glucoside
5	Ursolic Acid	57	Butein

NO.	English Name of the Compound	NO.	English Name of the Compound
6	Vanillic Acid	58	P-Hydroxy-Cinnamic Acid
7	L-Isoleucine	59	Liquiritin Apioside
8	Coumaric Acid	60	Protocatechuic Acid
9	Scopoletin	61	Quinic Acid
10	Adenosine	62	7-Hydroxy-4H-Chromen-4-One
11	Aurantiamide Acetate	63	Caffeic Acid
12	(1R,3R,4S,5S)-4-{[(2E)-3-(3,4-Dihydroxyphenyl)-2-Propenoyl]Oxy}-1,3,5-Trihydroxycyclohexanecarboxylic Acid	64	Chlorogenic Acid
13	Caffeate	65	Fa 18:1+3O
14	5-Hydroxymethyl-2-Furancarboxaldehyde	66	Narirutin
15	Quercetin	67	Methyl Eugenol
16	(2S,3S,4S,5R,6S)-3,4,5-Trihydroxy-6-(5-Hydroxy-4-Oxo-2-Phenylchromen-7-Yl)Oxyoxane-2-Carboxylic Acid	68	Asperulosidic Acid
17	Bergenin	69	(E)-3-(4-Hydroxy-3-Methoxyphenyl)Prop-2-Enoic Acid
18	2-Cyclohexen-1-One, 4-Hydroxy-4-(3-Hydroxybutyl)-3,5,5-Trimethyl-	70	Rutin
19	Dibutyl Phthalate	71	Beta-D-Galactose
20	Arginine	72	Phenylacetic Acid
21	D-Xylose	73	Quercetin-3-O-Galactoside
22	Palmitic Acid	74	Aconitic Acid
23	Beta-Eudesmol	75	Succinic Acid
24	Galactaric Acid	76	7,8-Dihydroxycoumarin
25	2-(Methylamino)Benzoic Acid	77	Alpha-D-Glucose
26	Norpseudoephedrine	78	Methyl Chlorogenate
27	4-Methoxycinnamic Acid	79	Naringenin-7-O-Glucoside
28	Hyperoside	80	Vanillin
29	Isovaleric Acid	81	Octadecanedioic Acid
30	Methylisoeugenol	82	Myo-Inositol

NO.	English Name of the Compound	NO.	English Name of the Compound
31	Kamebakaurin	83	Luteolin
32	Bisabolangelone	84	D-Mannose
33	Rupestonic Acid	85	Methyl Hexadecanoate
34	Scopoline	86	Kaempferol 3-O-Sophoroside
35	Asperuloside	87	L-Malic Acid
36	Kaempferol 3-O-Gentiobioside	88	Shikimic Acid
37	Anthranilate	89	(-)-Tracheloside
38	Curcumin I	90	Quercetin-3-O-(6''-Feruloyl)Sophoroside
39	Trans-4-Coumaric Acid	91	Quercetin 3-(6'''-Sinapylglucosyl)(1->2)-Galactoside
40	Hirsuteine	92	6-Hydroxyluteolin 7-O-[6'''-Benzoyl-Beta-D-Glucopyranosyl -(1->2)]-Beta-D-Glucopyranoside
41	Isoquercitrin	93	Methyl (3S,5S)-5-Hydroxy-3-(Beta-D-Glucopyran osyloxy)Hexanoate
42	Methyl Deacetylasperulosidate	94	Loganic Acid
43	Isoorientin	95	Kaempferol-3-O-Glucoside
44	Epigallocatechin-3-Gallate	96	Jasminoside D
45	Azuleno(5,6-C)Furan-1(3H)-One, 4,4A,5,6,7,7A,8,9-Octahydro-3,4,8-Trihydroxy-6,6,8-Trimethyl-	97	Germacrone
46	3-(2-Methylpropanoyloxy)-8-(3-Methylbutanoyloxy) -9,10-Epoxy-P-Mentha-1,3,5-Triene	98	Pterocarpan Base + 1O, 1Meo
47	3,4-Dimethylphenol	99	Vitamin C
48	Clareolide	100	Bufalin
49	7,8-Dihydroxyflavone	101	9Beta-Isobutyryloxyhelenalin Methacrylate
50	Jaceosidin	102	2Beta-Methoxy-6Alpha-O-Angeloyl-2,3-Dihydrohelenalin
51	17-Hydroxy-15,16-Epoxykauran-18-Oic Acid	103	1H-Pyrrole-2,5-Dicarboxylic Acid
52	Caryophyllene Epoxide	104	Telocinobufagin

Table S8. TCMSP screening of chemical constituents in *Hedyotis diffusa.*

NO.	Molecule Name	MW	AlogP	Hdon	Hacc	OB (%)
1	2,3-dimethoxy-6-methyanthraquinone	282.31	3.26	0	4	34.86
2	geniposide	388.41	-2.25	5	10	14.64
3	genipin	226.25	-0.50	2	5	26.06
4	2-hydroxy-3-methylanthraquinone	238.25	3.03	1	3	26.09
5	E-6-O-p-methoxycinnamoyl scandoside methyl ester	550.56	-1.04	6	13	27.12
6	E-6-O-p-methoxycinnamoyl scandoside methyl ester_qt	388.40	0.70	3	8	14.44
7	1H-2,6-dioxacyclopent(cd)inden-1-one, 4-((acetyloxy)methyl)-5-(beta-D-glucopyranosyloxy)-2a,4a,5,7b-tetrahydro-, (2aS-(2aalpha,5alpha,7balpha))-	414.40	-2.55	4	11	26.43
8	asperuloside_qt	252.24	-0.80	1	6	6.86
9	Oleanolic acid-28-O-beta-D-glucopyranoside	618.94	4.52	5	8	11.48
10	oleanolic acid-3-O-β-D-glucuronopyranoside_qt	456.78	6.42	2	3	17.93
11	(1S,4aS,5R,7aS)-5-hydroxy-7-methylol-1-[(2S,3R,4S,5S,6R)-3,4,5-trihydroxy-6-methylol-tetrahydropyran-2-yl]oxy-1,4a,5,7a-tetrahydrocyclopenta[d]pyran-4-carboxylic acid	390.38	-3.54	7	11	3.55
12	scandoside_qt	228.22	-1.79	4	6	30.02
13	3'-Hydroxyanethole	164.22	1.68	1	2	30.01
14	Poriferasterol	412.77	7.64	1	1	43.83
15	p-MCA	178.20	1.89	1	3	31.00
16	Scandoside methyl ester	404.41	-3.29	6	11	11.24
17	scandoside_qt	242.25	-1.54	3	6	14.41
18	(4aS,6aR,6aS,6bR,8aR,10R,12aR,14bS)-10-hydroxy-2,2,6a,6b,9,9,12a-heptamethyl-1,3,4,5,6,6a,7,8,8a,10,11,12,13,14b-tetradecahydropicene-4a-carboxylic acid	456.78	6.42	2	3	32.03
19	deacetylasperulosidic acid	390.38	-3.54	7	11	3.42
20	deacetylasperulosidic acid _qt	228.22	-1.79	4	6	30.29
21	Deacetyl asperulosidic acid methyl ester	404.41	-3.29	6	11	4.29

NO.	Molecule Name	MW	AlogP	Hdon	Hacc	OB (%)
22	deacetyl asperuloside acid_qt	242.25	-1.54	3	6	62.46
23	Geniposidic acid	374.38	-2.50	6	10	19.59
24	geniposidie acid_qt	212.22	-0.76	3	5	30.96
25	2-methoxy-3-methyl-9,10-anthraquinone	252.28	3.28	0	3	37.83
26	Digitolutein	268.28	3.01	1	4	19.36
27	Stigmasterol	412.77	7.64	1	1	43.83
28	succinic acid	118.10	-0.41	2	4	29.62
29	Sitogluside	576.95	6.34	4	6	20.63
30	beta-sitosterol	414.79	8.08	1	1	36.91
31	FER	194.20	1.62	2	4	39.56
32	Scopoletol	192.18	1.62	1	4	27.77
33	rutin	610.57	-1.45	10	16	3.20
34	ursolic acid	456.78	6.47	2	3	16.77
35	darutoside	574.93	5.89	4	6	21.32
36	p-coumaric acid	164.17	1.64	2	3	43.29
37	quercetin	302.25	1.50	5	7	46.43

Table S9. Relative expression levels of the relevant genes（$2^{-\Delta\Delta Ct}$，mean ± SD）.

Group	SPHK1	RELA	NFKBIA
Control	0.713 ± 0.080	0.790 ± 0.182	1.577 ± 0.445
ALI	0.930 ± 0.261	0.760 ± 0.139	1.120 ± 0.173
DXMS	0.750 ± 0.271	0.247 ± 0.245$^{\#*}$	1.193 ± 0.116
HDWE-H	0.577 ± 0.293	0.323 ± 0.204#*	1.883 ± 0.345$^{\#}$
HDWE-L	0.527 ± 0.127	0.427 ± 0.133	1.773 ± 0.214$^{\#}$

*P < 0.05 **P < 0.01 vs control group；$^{\#}P$ < 0.05 $^{\#\#}P$ < 0.01 vs model group.
